# Regeneration of iron species for high and stable activity of nickel electrodes in the oxygen evolution reaction†

**DOI:** 10.1039/d5gc00114e

**Published:** 2025-04-11

**Authors:** Stefano Poli, Claude Poleunis, Matteo Miola, Dominic Gerlach, Petra Rudolf, Arnaud Delcorte, Hans Lammers, Matheus T. de Groot, Dulce M. Morales, Paolo P. Pescarmona

**Affiliations:** a Chemical Engineering Group, Engineering and Technology institute Groningen (ENTEG), University of Groningen The Netherlands. E-mail: p.p.pescarmona@rug.nl; b Institute of Condensed Matter and Nanosciences – Bio & Soft Matter, Surface Characterisation, Université Catholique de Louvain Louvain-la-Neuve Belgium; c Zernike Institute for Advanced Materials, University of Groningen The Netherlands; d Nobian Van Asch van Wijckstraat 53 Amersfoort The Netherlands; e HyCC B.V. Van Asch van Wijckstraat 53 Amersfoort The Netherlands; f Department of Chemical Engineering and Chemistry, Sustainable Process Engineering Group, Eindhoven University of Technology The Netherlands

## Abstract

To enable green hydrogen production through alkaline water electrolysis, it is crucial to enhance the activity of nickel electrocatalysts towards the oxygen evolution reaction (OER), while preserving high stability. Here, we present a new and effective strategy to achieve this target through the introduction of short, periodical regeneration steps, complemented with the accurate tuning of traces of iron in the electrolyte. This strategy allowed retaining the enhanced activity brought about by the iron species adsorbed on the nickel electrode for the whole test duration (72 h) at an industrially relevant current density of 300 mA cm^−2^ with a 1.0 M KOH electrolyte containing *ca.* 100 ppb of iron (mimicking a commercial electrolyte). Under the same conditions but without regeneration, a dramatic deactivation was observed after *ca.* 18 h. Time-of-flight secondary ion mass spectrometry (ToF-SIMS) highlighted that such deactivation is correlated to the loss of iron species from the surface of the electrode. The regeneration steps help retain the iron species on the surface of the nickel electrode, thus granting the desired high OER activity and stability. We estimated that this regeneration strategy could lead to up to 18% energy saving compared to the current standard operating conditions of alkaline electrolysers.

## Abstract 2

A regeneration strategy combined with the tuning of traces of iron in the electrolyte enabled achieving high activity and enhanced stability of nickel electrodes in the oxygen evolution reaction (OER).

## Graphical abstract

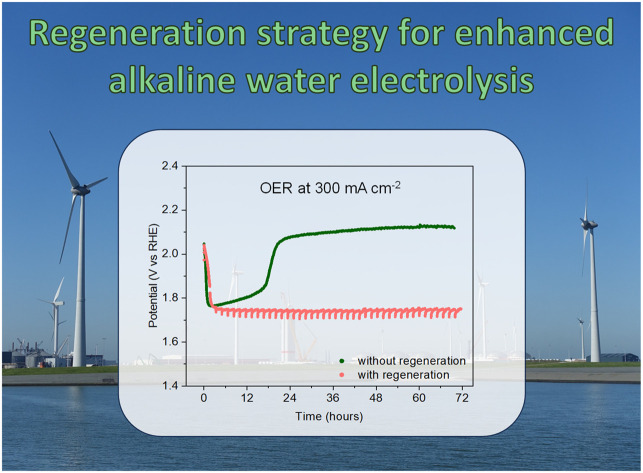

**Green foundation**

1. The production of green hydrogen through the electrolysis of water using renewable energy sources is key in the transition to a sustainable society. However, to make green hydrogen cost competitive it is crucial to improve the efficiency of the oxygen evolution reaction (OER). Here, we present a new strategy based on short, periodical regeneration steps, complemented with the tuning of traces of iron in the KOH electrolyte, which allows enhancing the activity of nickel electrocatalysts for the OER while granting high stability.

2. We estimated that our strategy could lead to up to 18% energy saving compared to the standard operating conditions of alkaline electrolysers.

3. Future research should aim at applying and, if necessary, adapting this strategy for alkaline water electrolysis in a flow cell, thus bringing it one step closer to commercial electrolysers.

## Introduction

Alkaline water electrolysis (AWE) has been an established technology for the production of hydrogen (H_2_) for more than a century,^1^ and yet it is currently not widely applied because the production of hydrogen from fossil resources (namely steam reforming of methane) is more cost-competitive.^2^ However, the finite nature of fossil resources and the CO_2_ emissions that stem from their utilisation are stimulating the scientific community to increase the research efforts to make the intrinsically sustainable production of hydrogen from water electrolysis also economically attractive. To underline the sustainable character of this route, which only relies on (potentially) renewable resources such as water and electric power,^3^ the hydrogen produced in this way is referred to as green hydrogen. Water electrolysis is not only a more sustainable way to produce H_2_ as a bulk chemical, but it can also provide a solution to the intermittent nature of renewable power sources (*e.g.* wind, solar and tidal energy) by enabling storage of the electricity that cannot be immediately utilised into hydrogen as an energy carrier. Therefore, improving the economic feasibility of water electrolysis technology is a target of crucial industrial and societal relevance. There are several technological challenges to be overcome to increase the cost-competitiveness and thus make alkaline water electrolysis more accessible to the market, ranging from the design of the electrolysers to the efficiency and durability of their main components, *i.e.* the electrodes and separators.^4^

**Fig. 1:**
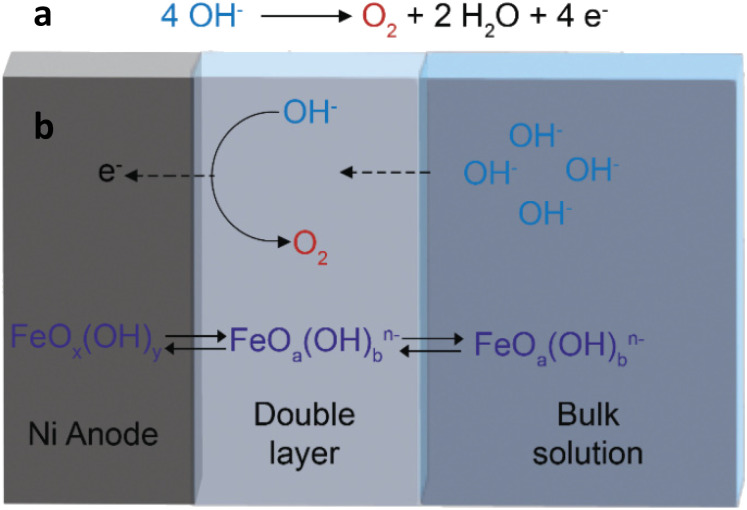
(a) The oxygen evolution reaction (OER) occurring at the anode of a water electrolyser in an alkaline environment. (b) Schematic representation of the main mass transfer phenomena involving dissolution/redeposition of iron species stemming from the electrolyte, occurring during the OER on a Ni electrode.

At the industrial scale, water electrolysis is carried out in stacks consisting of several electrochemical cells operating simultaneously. In each of these cells, the hydrogen evolution reaction (HER) takes place at the cathode and is coupled with the oxygen evolution reaction (OER) that occurs at the anode. Although the target product of water electrolysis (H_2_) is generated through the HER, the OER is the most challenging step of the overall process because this half-reaction requires the transfer of 4 electrons to form one O_2_ molecule (Fig. 1a), compared to the 2 electrons exchanged in the formation of one H_2_ molecule, and thus generally involves a significantly higher overpotential.^2^ Developing efficient electrocatalysts that minimise the overpotential for the OER is essential to make industrial water electrolysers cost-competitive, since lower overpotentials would lead to a decrease in the overall power demand, thus decreasing the operating expenses (OPEX). Against this backdrop, major research efforts are currently dedicated to the discovery of novel electrodes for the OER with enhanced performance in terms of activity and durability.^5–10^ When taking into account the target of large scale application of water electrolysers, the abundance and cost of the metal(s) constituting the anode are additional important parameters to be considered. In this context, the relatively widely available Ni is currently the industrial benchmark as an anode material for water electrolysers operating in an alkaline environment.^6,11^ However, some aspects of the electrocatalytic behaviour of nickel electrodes are not yet fully elucidated, especially when in combination with iron species. The addition of iron, even at the impurity level (≤100 ppb), has been demonstrated to greatly boost the activity of nickel electrodes towards the OER.^12–14^ Iron can be deliberately added to nickel electrodes, but it has also been shown that iron impurities from the electrolyte can be adsorbed and incorporated while the cell is idle^13^ or when a potential is applied.^12,15^ It has been shown that iron is not only deposited on the surface of the anode, but also gets depleted from it by leaching into the electrolyte during electrolysis.^16^ The loss of iron has been proposed to be the cause of the deactivation of the electrode during the OER that is generally observed with Fe-containing Ni electrodes.^16–19^ A recent study indicated that the concentration of iron in the electrolyte solution affects the balance between the dissolution and reprecipitation rates of the iron species, and this in turns determines the stability of NiFe electrocatalysts.^18^ Here, we present a novel strategy to regenerate the iron species in Ni electrodes during the OER, thus combining high activity and enhanced stability. The strategy aims at limiting the depletion of the surface iron species that are responsible for the improved activity of the nickel anodes. We reasoned that decreasing periodically and for short time the potential from the one at which the OER occurs could help redeposit oxidised iron species that leach from the nickel electrode surface into the electrolyte during the OER (Fig. 1b). Our regeneration approach proved effective in retaining the high OER activity of Ni electrodes brought about by iron species that are present as impurities in the commercial KOH electrolyte, leading to an improvement (*i.e.* a decrease) of up to 380 mV in the potential needed to sustain an industrially relevant current density of 300 mA cm^−2^. The influence of the presence of iron at the surface of the nickel electrode was elucidated by time-of-flight secondary ion mass spectrometry (ToF-SIMS) and X-ray photoelectron spectroscopy (XPS). The fact that our regeneration strategy can be directly utilised with Ni electrodes similar to those used industrially and with a commercial KOH electrolyte can pave the way to a possible straightforward application in the current electrolyser technology, and is thus highly promising for increasing the competitiveness of alkaline water electrolysis against other electrochemical^20^ and chemical^21^ routes for the production of hydrogen.

## Results and discussion

### Regeneration of the activity of Ni-based electrodes

In this work, we aimed at improving the performance of the most commonly used anodes for alkaline water electrolysis, which consist of, or include, nickel,^5–7,22^ by means of a tailored regeneration approach aimed at preserving the iron species at the electrode surface during the OER. As the starting material for our experiments, we chose a nickel wire (99.995% Ni) as this is a representative of the benchmark electrodes for the industrial alkaline OER that consist of nickel either as perforated plates or meshes.^23^

**Fig. 2:**
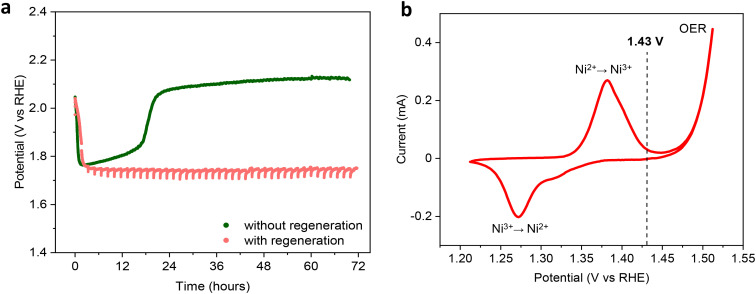
(a) Chronopotentiometry of a nickel wire electrode at 300 mA cm^−2^ with and without regeneration steps in 1.0 M commercial KOH at 30 °C. During regeneration, the electrode potential is decreased to 1.43 V for 100 s every 100 min of electrolysis, for a total time of electrolysis of 72 h. The potential is *iR* compensated. (b) Cyclic voltammetry of a nickel wire (10 mV s^−1^). The vertical line at 1.43 V corresponds to the potential used for the regeneration step in the chronopotentiometry.

Initially, we assessed the OER performance of a bare nickel wire in a chronopotentiometric test at an industrially relevant current density of 300 mA cm^−2^ for 72 h in a 1.0 M commercial KOH electrolyte solution (see the Methods section for more details). Under these conditions, the nickel electrode initially showed an activation, as demonstrated by the sharp decrease in the applied potential needed to sustain 300 mA cm^−2^, which decreased from the initial 2.05 to 1.75 V *vs.* RHE within the first few minutes of electrolysis (Fig. 2a). The nickel anode then displayed a gradual, slow loss of activity during the subsequent 18 h of the OER, followed by a more dramatic, sudden deactivation between 18 and 22 h that led to an increase of the potential to a value above the initial 2.05 V *vs.* RHE, after which the performance remained nearly constant for the rest of the experiment. We reasoned that the initial activation was related to the adsorption of iron impurities from the electrolyte, and the following deactivation was caused by their gradual release back to the electrolyte and/or to the cell. To counter this deactivation, we envisaged a regeneration procedure, in which the applied potential was lowered to 1.43 V *vs.* RHE for 100 s every 100 min of electrolysis (*i.e.* the regeneration time corresponds to 1.67% of the electrolysis time). The potential chosen for these regeneration steps was well below the one at which the anode is operated during the OER, and just above the Ni(ii)/Ni(iii) potential (Fig. 2b), to avoid the reduction of Ni(iii) to Ni(ii). The latter is a crucial feature, as reduction of Ni(iii) at the anode can cause concomitant oxidation of the Ni-based cathode generally used in alkaline electrolysers, and this may accelerate its degradation.^24,25^

Our strategy proved very successful and led to significantly higher stability in the OER performance; also in this case, the nickel electrode quickly activated after a few minutes of electrolysis, but then maintained a practically constant activity within the 72 h of the test, with the applied potential needed to sustain 300 mA cm^−2^ remaining below 1.75 V (see Fig. 2a). This corresponds to a *ca.* 380 mV lower potential needed to sustain the target current density by the end of the test compared to the experiment without regeneration. Preliminary regeneration tests for 24 h at other potential values (*i.e.* open circuit potential, 1.23 *vs.* RHE and CVs across the Ni(ii)/Ni(iii) redox peak) worked similarly well in preserving the activity of the Ni anode (Fig. S1†). However, since the potential of 1.43 V *vs.* RHE is expected to be preferable for the stability of the Ni cathodes used in AWE, we selected it for the rest of this study.

An estimation of the electrical energy utilised in the two experiments reported in Fig. 2 shows that the regeneration allows a ∼14% decrease of the energy input over the 72 h electrolysis test. The energy saving increases to ∼18% if we mimic the impact that the regeneration would have on long-term electrolysis by comparing the two experiments in the range between 24 and 72 h, *i.e.* after deactivation occurred in the test under conventional conditions (see ESI Note 1† for the calculations). It is worth reporting that regeneration of nickel electrodes by decreasing the applied potential was previously observed by Mellsop *et al.*, though under significantly different conditions (50 mA cm^−2^, 30 wt% KOH).^26^ However, the authors correlated the activation to the conversion of Ni(OH)_2_ to more active nickel oxyhydroxides (NiOOH) and attributed the successive deactivation to the formation of Ni(iv) species on the electrode surface,^26^ while not considering the possible role of the iron species. Our next experiments were thus aimed at testing our hypothesis of the influence of iron on the activation/deactivation and on the regeneration of Ni electrodes.

Electrochemical studies are often plagued by a low degree of reproducibility. Therefore, we took special care in checking and where necessary improving the reproducibility of our results. The experiments shown in Fig. 2 were highly reproducible (Fig. S2†) when the same batch of commercial KOH was used to prepare the electrolyte, though we did observe variations between KOH batches. This is most likely related to different contents of iron impurities in different batches of commercial KOH. We achieved a better control on the electrochemical tests by purifying the commercial KOH electrolyte from iron, followed by the intentional addition of well-defined amounts of iron (the procedure is described in the Methods section). This allowed us to control the amount of iron in the electrolyte and thus to elucidate the relation between the regeneration approach and the iron species on Ni electrodes.

### Influence of the iron concentration in the electrolyte

**Fig. 3:**
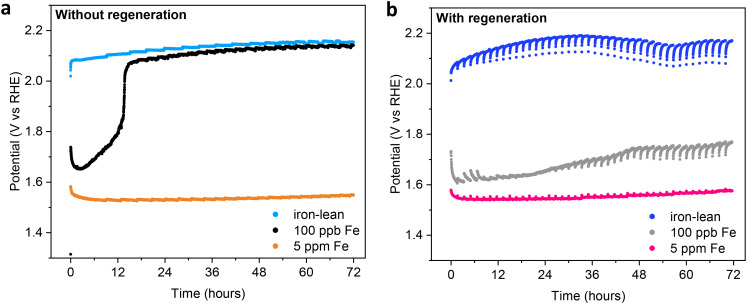
Chronopotentiometry at 300 mA cm^−2^ for 72 h at 30 °C of nickel wire electrodes without (a) and with (b) regeneration, in an iron-lean electrolyte, iron-lean electrolyte with 100 ppb Fe intentionally added (to mimic the iron content of a commercial 1.0 M KOH electrolyte), and iron-rich electrolyte (5 ppm Fe). The potential is *iR* compensated.

To gain a deeper understanding of the way in which our regeneration strategy helps preserve the OER activity of Ni electrodes, we investigated the regeneration behaviour as a function of the concentration of iron in the electrolyte. For this purpose, the commercial 1.0 M KOH solution used as the electrolyte was meticulously purified using a literature procedure (expected to lead to an iron concentration <36 ppb).^12^ This, in combination with the high purity of the nickel wire that we employed as the working electrode, allowed us to perform tests under iron-lean conditions, in which the nickel electrode displayed very poor OER activity as indicated by the high potential (>2.10 V *vs.* RHE) required to sustain the target current density of 300 mA cm^−2^ (Fig. 3a, light blue curve). This confirms the influence of iron species on the activity of Ni electrodes previously demonstrated by Trotochaud *et al.*^12^ When the regeneration strategy was applied to this system, no significant change in the performance of the Ni electrode was observed (Fig. 3b, blue curve).

Next, we prepared an electrolyte with a nominal concentration of 100 ppb Fe by controlled addition of Fe(NO_3_)_3_ to the iron-lean electrolyte. This concentration of iron was chosen as it is expected to be in the range of the iron content of 1.0 M commercial KOH electrolyte, and thus to allow mimicking its behaviour in a controlled way. In line with our expectations, the nickel wire with 100 ppb Fe addition in the electrolyte displayed a similar electrocatalytic behaviour to the one observed with commercial KOH (compare Fig. 2a and 3a), with an initial activation to 1.65 V *vs.* RHE, followed by a rather sudden and quick deactivation to 2.05 V *vs.* RHE after *ca.* 13 h of electrolysis. It is worth noting that the potential after deactivation is very similar to that observed in the test with the iron-lean electrolyte (compare blue and black curves in Fig. 3a), suggesting that the deactivation is correlated to the loss of iron from the electrode surface. When the test was carried with the regeneration steps, the trend was again similar to that observed with the commercial KOH electrolyte, with the electrocatalytic activity of the Ni electrode being largely preserved throughout the 72 h test (grey curve in Fig. 3b). The striking difference in the effect of our regeneration strategy in the iron-lean test compared to the one with 100 ppb Fe clearly proves that the regeneration is closely correlated to the iron species in the electrolyte.

**Fig. 4:**
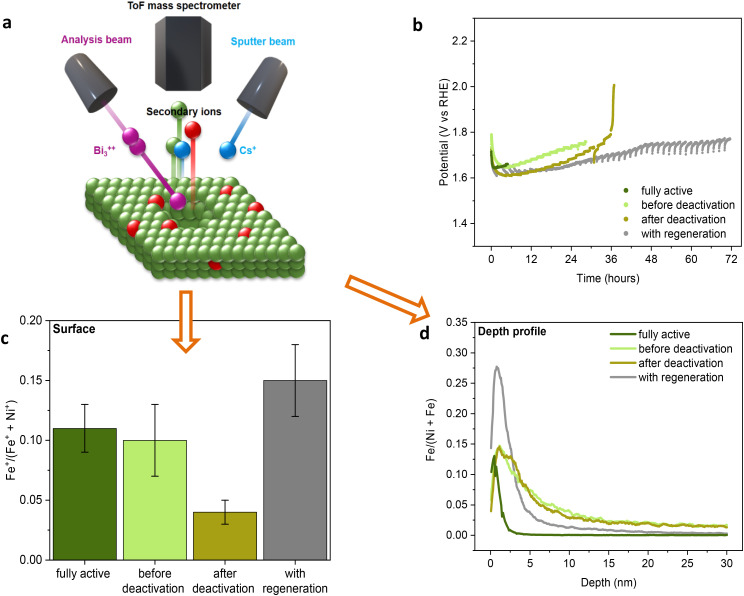
(a) Schematic representation of the ToF-SIMS working principle. (b) Chronopotentiometry at 300 mA cm^−2^ of a nickel wire electrode without or with regeneration steps (1.43 V for 100 s every 100 min of electrolysis) in iron-lean 1.0 M KOH at 30 °C with 100 ppb Fe intentionally added. Samples were collected at different moments during the experiment without regeneration, while the sample with regeneration was collected after 72 h of electrolysis. The potential is *iR* compensated. (c) Surface iron content based on ToF-SIMS measurements (area 150 μm × 150 μm) and (d) ToF-SIMS depth-profiling of iron (area 450 μm × 450 μm) of the samples from the chronopotentiometric test in (b). Fe and Ni relative contents calculated from the sum of M^+^, CsM^+^ and Cs_2_M^+^ fragments, where M = Fe or Ni.

To support this conclusion, ToF-SIMS was employed to determine the amount and the location (surface *vs.* subsurface) of iron in three nickel electrodes with different degrees of deactivation during the chronopotentiometric OER test without regeneration (300 mA cm^−2^ in an electrolyte solution of 1.0 M KOH at 30 °C containing 100 ppb Fe(iii)), as shown in Fig. 4. These electrodes are hereafter referred to as “fully active” (collected when the electrode displayed the highest activity), “before deactivation” (collected right before the inflection point corresponding to the sharp increase in potential) and “after deactivation” (collected right after the inflection point). For comparison, a fourth nickel electrode was tested under the same conditions but with the regeneration steps. ToF-SIMS measurements of these samples allowed us to monitor the presence of iron by sputtering the surface (depth of analysis of *ca.* 2 nm) of the sample with Bi_3_^++^ ions, followed by the analysis of the extracted fragments on a time-of-flight mass spectrometer. This technique is advantageous compared to other characterisation techniques (*e.g.* XPS) due to its lower detection limit. In the absence of the regeneration steps, ToF-SIMS clearly showed that iron species are progressively depleted from the surface of the nickel electrode during the OER (Fig. 4c). The loss of iron is minor from the fully active electrode to the one that only experienced a small, gradual deactivation; whereas a major loss of iron is observed after the sudden, major deactivation corresponding to the inflection point in the chronopotentiometric plot (Fig. 4b and c). On the other hand, when the regeneration strategy was used, the nickel wire largely retained its activity for 72 h and at the end of the test the relative iron content on its surface was much higher compared to the deactivated electrode. These results strongly support the correlation between the loss of surface iron species and the deactivation of the electrode, and the role of the regeneration steps in preventing iron depletion from the electrode surface.

The surface composition of the same electrode samples was also analysed by XPS (Fig. S3–S8†). The Ni 2p signals indicated the expected gradual oxidation of surface nickel species during electrolysis. However, no significant correlation was observed between the deactivation of the electrode and the oxidation state of the surface nickel species, with the Ni 2p signals before and after deactivation being nearly identical (Fig. S3†). XPS data are not very informative for monitoring the iron content, as the detection of the Fe 2p signal is hindered by the overlapping Auger line of Ni (Fig. S4†). Yet, a very low-intensity Fe 3p signal was observed in the fully active electrode, while no Fe signal was detected for the fresh electrode and for the deactivated electrode (Fig. S5†), thus supporting the trends observed by ToF-SIMS.

In order to have a better understanding of the fate of the iron species that are lost from the electrode surface if the OER is carried out without the regeneration steps, the depth profile of the metal composition was measured by sputtering the surface with a secondary Cs^+^-beam during the ToF-SIMS analysis (Fig. 4d). The nickel wire collected after 5 h of electrolysis without regeneration (but still “fully active”) showed negligible iron content in the subsurface region at a depth >4 nm. On the other hand, the Ni wires just before and just after the deactivation in the experiment without regeneration are richer in iron in the subsurface area compared to the fully active wire (Fig. 4d), though they are leaner in Fe in the surface region (Fig. 4c). The depth profile of the Ni wire tested for 72 h with regeneration shows that this electrode is the richest in Fe in the surface and near-surface regions, whereas in the subsurface region at a depth >4 nm, the iron content is lower compared to the deactivated electrode in the test without regeneration (Fig. 4c and d). These results suggest that the deactivation in the electrolysis without regeneration might be attributed not only to loss of iron into the electrolyte solution^16^ but also to its incorporation into the subsurface region of the electrode, possibly due to redeposition and surface reconstruction processes that have been reported to occur during the OER.^16^ The fact that electrodes displaying high OER activity are richer in Fe on the surface but leaner in the subsurface region compared to the deactivated electrodes is in agreement with previous reports proposing that only the superficial Fe species, which are not fully coordinated (*e.g.* on edges or defects), promote the oxygen evolution reaction.^15^ This may also explain why there is still no agreement on the optimal Ni–Fe composition of OER electrodes (ranging from as low as 10% Fe, up to 40% Fe ^27–30^), since the amount of iron available on the surface may vary according to the synthesis method and the conditions adopted for the preparation of the electrodes.

Once the effect of traces of iron from the electrolyte and the importance of our regeneration approach in preserving them at the Ni electrode surface were ascertained, we extended our study by testing the effect of carrying out the OER with an iron-rich electrolyte containing 5 ppm of Fe(iii). When this high concentration of iron was used in the electrolyte, the OER activity of the bare Ni wire was strongly enhanced, as shown by the decrease of approximately 0.6 V in the applied potential needed to operate the cell at 300 mA cm^−2^ compared to the test with the iron-lean electrolyte (Fig. 3). It is worth noting that a red precipitate gradually formed at the bottom of the cell from the electrolyte containing 5 ppm Fe, and the solid persisted for the whole duration of the experiment, which indicates that the electrolyte was (over)saturated with iron(iii) (oxy)hydroxides. In the presence of this higher concentration of iron, the electrode also showed high stability, with the applied potential needed to sustain a current density of 300 mA cm^−2^ remaining below 1.60 V *vs.* RHE for the full 72 h of the chronopotentiometric test. Notably, in the tests with 5 ppm Fe, the electrode performance remained stable both with or without the regeneration steps (compare the orange curve in Fig. 3a with the magenta curve in Fig. 3b).

**Fig. 5:**
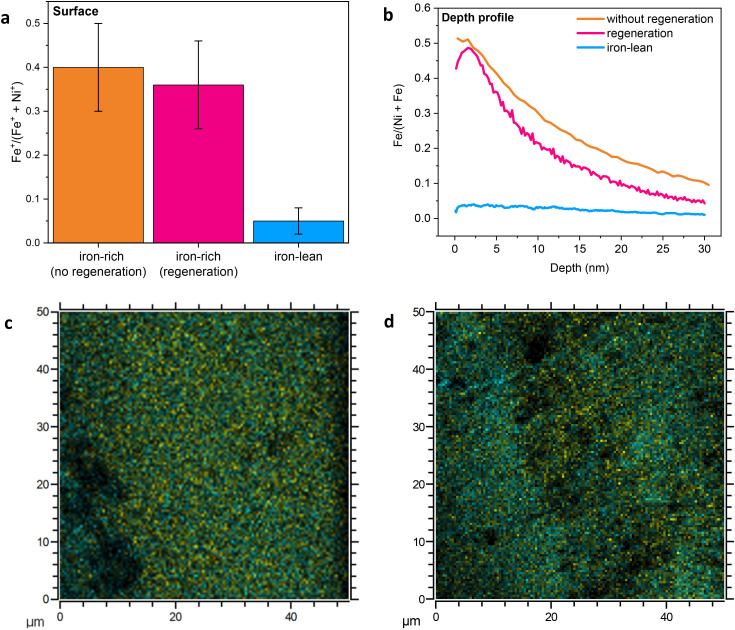
(a) Surface iron content based on ToF-SIMS measurements (area 150 μm × 150 μm) and (b) ToF-SIMS depth-profiling of iron (area 450 μm × 450 μm) of the electrodes after the chronopotentiometric tests at 300 mA cm^−2^ for 72 h with the iron-rich 1.0 M KOH electrolyte (5 ppm Fe), with or without regeneration (the chronoamperometry values of these electrodes are shown in Fig. 3a and b). The Fe and Ni relative contents in the depth profile were calculated from the sum of M^+^, CsM^+^ and Cs_2_M^+^ fragments, where M = Fe or Ni. The ToF-SIMS data for the surface iron content and the depth profile of the electrode after the chronopotentiometric tests at 300 mA cm^−2^ for 72 h with the iron-lean 1.0 M KOH electrolyte without regeneration are provided for comparison (the chronoamperometry of this electrode is shown in Fig. 3a). (c and d) ToF-SIMS elemental mapping of the surface of the same two electrodes after the chronopotentiometric test with the iron-rich electrolyte, with (c) and without (d) regeneration. Yellow is the total sum of positively charged iron-containing fragments (^54^Fe^+^, Fe^+^, FeH^+^, FeOH^+^) and cyan is the total sum of positively charged nickel-containing fragments (Ni^+^, NiH^+^, ^60^Ni^+^, ^60^NiH^+^, ^60^NiH^2+^); see Fig. S9† for the separate mapping for iron and nickel. For the mapping of other elements, see Fig. S10 and 11.†

These findings were corroborated by ToF-SIMS analysis of the surface and subsurface iron content of the nickel wire electrodes after the 72 h electrolysis test in the iron-rich electrolyte, with and without regeneration (Fig. 5). The surface Fe contents of the two samples were similar, and in both cases much higher compared to those of the nickel wires that were tested in the iron-lean electrolyte and with 100 ppb of Fe (compare Fig. 4c with Fig. 5a). This indicates that the iron-rich electrolyte ensures a high Fe content on the surface of the electrode, which is preserved in the explored timeframe irrespective of the use of the regeneration approach. These results further support the correlation between the presence of iron at the surface of the Ni-based electrode and its OER activity. Depth-profile measurements of these two nickel wire electrodes reveal that a significant amount of iron was incorporated into the subsurface region of the electrodes during electrolysis (Fig. 5b). Similarly to what was observed with the samples tested with 100 ppb Fe in the electrolyte (Fig. 4c), the nickel wire electrode with regeneration showed a lower Fe content in the subsurface region than the sample without regeneration (Fig. 5b), suggesting that the regeneration steps limit the accumulation of iron in the subsurface of the electrode. ToF-SIMS also allowed mapping of the surface distribution of Fe and Ni in an area of 50 μm × 50 μm for the two nickel wire electrodes tested with the iron-rich electrolyte. This analysis showed that the distribution of iron and nickel is rather uniform after the electrolysis test involving the regeneration steps (Fig. 5c), whereas domains richer in each of the two elements are observed after the test carried out without regeneration (Fig. 5d). It has been reported that iron tends to segregate from nickel during long and continuous OER experiments due to a dissolution–redeposition mechanism, and this phenomenon was correlated with the deactivation of the electrode.^17^ Since our regeneration strategy helps maintain a uniform distribution of iron and nickel, it can be expected that while the two electrodes tested with the iron-rich electrolyte with or without regeneration displayed similar, high and constant OER activity during 72 h of electrolysis at 300 mA cm^−2^, on the longer term the regeneration would help preserve the activity further, similarly to what we observed with the electrolyte containing 100 ppb of iron.

**Fig. 6:**
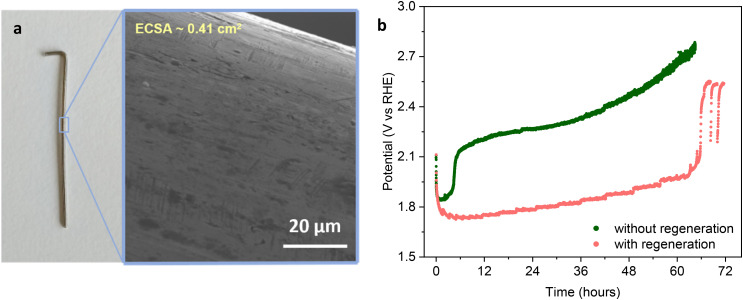
(a) A fresh nickel wire and a SEM image of its surface. (b) Chronopotentiometry at 800 mA cm^−2^ for 72 h of bare nickel electrodes with and without regeneration, with 120 ppb Fe added to an iron-lean 1.0 M KOH electrolyte. The experiments were carried out at 30 °C. The potential is reported *vs.* RHE and it is *iR* compensated. The regeneration steps were performed by lowering the applied potential to 1.43 V for 100 s every 100 min of electrolysis.

By comparing the results obtained with the iron-lean electrolyte with those obtained with the electrolyte containing 100 ppb Fe and 5 ppm Fe, and the different effects that the regeneration steps have in each of these three cases, it can be concluded that the iron species that lead to enhanced OER activity of Ni-based electrodes by being deposited on their surface tend to get depleted during electrolysis, with the effect being more significant if the concentration of iron in the electrolyte is low (100 ppb). The characterisation by ToF-SIMS proved that the loss of iron from the electrode surface is the cause of the observed deactivation. This issue can be minimised by using our regeneration steps, which limit the loss of the Fe species from the electrode surface. With the iron-rich electrolyte (5 ppm), the high activity is preserved for the whole 72 h of the chronopotentiometric test also without the regeneration steps, due to the abundant supply of Fe. However, a more uniform distribution of iron and nickel was observed in the test with regeneration, and this may help prevent segregation leading to loss of activity on the longer term. Additionally, it is worth noting that iron (oxy)hydroxides that tend to precipitate from the highly oversaturated electrolyte containing 5 ppm of iron in 1.0 M KOH might lead to membrane fouling and/or cathode contamination, and thus be a limitation for the use of iron-rich electrolytes. For these reasons, the combination of trace amounts of iron in the electrolyte with the regeneration approach might be preferable in the perspective of large-scale application. In this context, we explored whether our strategy is effective also at a competitively high current density of 800 mA cm^−2^ (Fig. 6). It should be highlighted that this is an extremely high current density for an anode that has a very low roughness as the Ni wire used as the model electrode in this study (see the SEM image in Fig. 6a). To quantify this feature, we estimated the electrochemically active surface area (ECSA) of a Ni wire (*ca.* 1.2 cm length, Fig. 6a) by linear sweep voltammetry (LSV), obtaining a value of 0.41 cm^2^ for a geometric surface area (*A*_geo_) of *ca.* 0.19 cm^2^, corresponding to a roughness factor ECSA/*A*_geo_ = 2.16. This implies that for the Ni wire, a current density of 800 mA cm^−2^_geometric area_ corresponds to a current density of 371 mA cm^−2^_ECSA_. As a comparison, it is instructive to note that state-of-the-art nanostructured Ni-based electrodes have been reported to display an ECSA that is up to two orders of magnitude larger than that of the Ni wire used in this work.^31^ As a consequence, the current density per ECSA unit (expressed in mA cm^−2^_ECSA_) experienced by the Ni wire is extremely large, making the conditions of this test much more challenging compared to those to which a nanostructured electrode is subjected in a chronoamperometric test at the same current density relative to the geometric area.

In the test at 800 mA cm^−2^, 120 ppb of iron was added to 1.0 M KOH electrolyte solution and the regeneration procedure was kept the same as for the other experiments at a lower current density. In the test without regeneration, the electrode deactivated after only 2 h of electrolysis with a steep increase of the potential needed to achieve the target current density from *ca.* 1.85 to >2.15 V *vs.* RHE, after which the potential kept increasing gradually reaching values >2.70V (Fig. 6b). On the other hand, when the regeneration was applied, the potential only very slowly and gradually increased while remaining at <1.85 V *vs.* RHE for the first 36 h. Under the more extreme conditions experienced by the anode in this test, deactivation eventually occurred after 66 h of electrolysis, as evidenced by the sudden increase of the potential to values >2.50 V *vs.* RHE (Fig. 6b). These results further prove the impact that our strategy has on improving the activity and extending the stability of the bare nickel wire electrode, while they also indicate that the specific regeneration conditions and minimum iron content needed to maintain a stable, low potential depend on the target current density. Future work will aim at optimising these conditions to achieve stable performance also at this very high current density.

The Ni wire electrodes recovered after the tests discussed above were analysed by SEM. Compared to the relatively smooth surface of the pristine wire (Fig. 6a), the surface of the used electrodes showed hills with a size between a few hundred nm up to the μm-scale (Fig. S12–18†). Notably, the formation of these hills was much more marked for the electrodes that suffered deactivation and was only minor for the electrodes that retained their activity. We hypothesise that the formation of these hills occurs upon surface dissolution and reprecipitation that is promoted by the high potential (>2 V *vs.* RHE) required for sustaining 300 mA cm^−2^ with the deactivated electrodes. Future studies will aim at understanding further this phenomenon.

Finally, we performed a control experiment in which the OER was carried out on a Ni wire electrode at 300 mA cm^−2^ for 1 h in an iron-lean KOH electrolyte, followed by the addition of 5 ppm of Zn(ii), and after another 1 h by the addition of 5 ppm of Fe(iii). The addition of 5 ppm of Zn(ii) had a negligible effect on the potential needed to sustain the current density of 300 mA cm^−2^, whereas the addition of 5 ppm of Fe(iii) led to a sudden activation of the electrode as indicated by the decrease in the potential from 2.04 to 1.62 V *vs.* RHE (Fig. S19†). This experiment supports the specificity of iron in enhancing the OER activity of nickel electrodes.

## Conclusions

This work introduces a straightforward yet highly effective strategy for improving the performance of nickel anodes for the oxygen evolution reaction (OER), which is the anodic reaction in the alkaline water electrolysis technology used to produce green hydrogen. Our study reveals that the loss of activity due to the depletion of active surface iron species (typically originating from impurities in the KOH electrolyte) can be largely or even fully prevented with a regeneration approach, consisting of lowering the applied potential to a value (1.43 V *vs.* RHE) that is just above the Ni(ii)/Ni(iii) peak and well below the OER onset, for 100 s every 100 min of electrolysis (*i.e.* for less than 2% of the electrolysis time). In the OER with a commercial 1.0 M KOH electrolyte containing trace impurities of iron, our regeneration strategy greatly improved the stability of nickel anodes, allowing the preservation of a nearly constant, high activity: an anodic potential of *ca.* 1.75 V *vs.* RHE allowed sustaining an industrially relevant current density of 300 mA cm^−2^ in a 72 h chronopotentiometric test. This is a striking improvement compared to the same test without regeneration, under which conditions the electrode suffered a major deactivation after approximately 18 h of electrolysis, eventually leading to a 380 mV higher potential after 72 h of electrolysis at 300 mA cm^−2^. We estimated that this regeneration strategy can lead to up to 18% energy saving for the OER at this industrially relevant current density.

The regeneration approach also proved effective in limiting the deactivation of nickel electrodes when 100–120 ppb of Fe was intentionally added to a purified iron-lean KOH electrolyte (mimicking the level of iron impurity in commercial KOH), not only at 300 mA cm^−2^ but also at the competitively high current density of 800 mA cm^−2^.

Characterisation of the surface and subsurface regions of the nickel electrode using ToF-SIMS showed that the deactivation of the electrodes under conventional operating conditions is correlated to the loss of surface iron species, either into the electrolyte or into the subsurface region of the electrode. The regeneration steps help retain the iron species on the surface of the nickel electrode, thus granting the desired high OER activity and stability. Since this regeneration occurs by temporarily decreasing the applied potential, it is reasonable to assume that it involves the redeposition of highly-oxidised iron species leached from the electrode surface. Future studies can aim at shedding more light on the specific nature of the leached species and on their role in the OER mechanism.

The fact that this study was carried out on a bare nickel electrode that resembles those used in the current alkaline water electrolysis technology, implies that our results can have a major impact on decreasing the power demand of alkaline water electrolysers, thus increasing their competitiveness for the production of green hydrogen. The next step in this sense will be to investigate how the interplay between regeneration and iron content highlighted by this work translates when applied in a flow cell that more closely resembles commercial electrolysers.

Finally, an additional attractive feature of our strategy is that under the regeneration conditions only a very low anodic current passes through the electrode (<50 μA cm^−2^). This means that these conditions might lend themselves to be adopted as the rest state of the electrolyser when low power input is available due to intermittency of the renewable energy sources. This would allow avoiding the reverse currents that occur upon shut-down of the electrolyser and which are detrimental for the lifetime of the cathode.^24^

## Experimental

### Electrochemical setup

The experiments were performed in a thermostated H-cell (Fig. S20 and 21†), operated in the 3-electrode configuration. Water was recirculated from the thermostat to the jacket of the cell in order to keep a constant temperature of the electrolyte of 30 °C. Selemion AHO was used as membrane separator between the anodic and cathodic compartments. In the 3-electrode configuration of the H-cell, the working electrode (WE) and the reference electrode (RE) were placed in the anodic compartment, and the counter electrode (CE) in the cathodic compartment. The working electrode was a nickel wire (99.995% Ni, 0.5 mm diameter, Puratronic, AlfaAesar). The wire was cut to a length of approximately 2.0–2.2 cm, so that the part immersed in the electrolyte during the experiment was *ca.* 1.2 cm long (∼0.19 cm^2^ geometric surface area), and it was sonicated for 15 min in ethanol followed by 10 min in MilliQ water to remove possible impurities introduced by the production process and handling. The reference electrode was Hg|HgO|KOH (1 M) supplied by ALS (0.098 V *vs.* SHE). All the values of the potential are reported *versus* RHE (see ESI Note 2† for the conversion formula). The counter electrode was a nickel mesh (nickel gauze, 100 mesh woven from 0.1 mm, AlfaAesar). See Fig. S20† for further details about the cell. The experiments were performed with a Gamry 1010E potentiostat.

### Electrolyte preparation

The electrolyte was a 1.0 M KOH (pH 13.8 at 30 °C) aqueous solution prepared by dissolving analytical grade KOH pellets (Supelco, declared iron content <0.0005%) in MilliQ water (16.2 MΩ). The electrolyte prepared in this way is referred to as commercial KOH electrolyte. The iron-lean KOH electrolyte was prepared starting from the commercial KOH electrolyte by following the purification procedure described by Trotochaud *et al.*^12^ In summary, the procedure involves the precipitation of Ni(OH)_2_ from Ni(NO_3_)_2_. Approximately 2 g of Ni(NO_3_)_2_ was dissolved in 4 mL of MilliQ water inside a 50 mL polypropylene (PP) centrifuge tube, to which 20 mL of 1.0 M KOH aqueous solution was then added to cause the precipitation of Ni(OH)_2_. Ni(OH)_2_ precipitated forming a powder, which was separated and recovered by centrifugation at 4000 rpm for 4 min, followed by removal of the supernatant (which was discarded). The obtained Ni(OH)_2_ (as a wet powder) was washed by adding 20 mL of MilliQ water and 2 mL of 1.0 M KOH solution to the centrifuge tube, redispersing the solid by shaking vigorously (manually, or if necessary in a vortex mixer) until a homogeneous suspension was achieved (*i.e.* with no visible chunks of Ni(OH)_2_), centrifuging as above and removing the supernatant. This step was repeated 3 times. The purified Ni(OH)_2_ thus prepared was subsequently used to remove the iron impurities from the commercial KOH electrolyte. For this purpose, approximately 45 mL of commercial KOH electrolyte was introduced in a centrifuge tube containing the previously prepared Ni(OH)_2_, and shaken vigorously (manually, or if necessary in a vortex mixer) until a homogeneous suspension was achieved. Then, the suspension was kept static for at least 4 h, flipping the tube every 2 h to redisperse any Ni(OH)_2_ that might have settled. Next, the suspension was allowed to decant overnight, and then centrifuged at 4000 rpm for 4 min to separate Ni(OH)_2_ by settling it at the bottom of the tube. The supernatant iron-lean KOH electrolyte was then either poured into the cell or into another clean PP centrifuge tube.

The iron-rich KOH electrolyte was prepared from the commercial KOH electrolyte by addition of 10 μL of an aqueous solution containing 18000 ppm of Fe^3+^ (*ca.* 0.32 M, prepared from Fe(NO_3_)_3_) to 36 mL of iron-lean KOH solution (previously added into a 50 mL PP centrifuge tube) and shaken vigorously (manually). The iron addition to the iron-lean electrolyte was performed prior to each experiment and the obtained solution was immediately poured into the anodic compartment (36 mL) of the H-cell. The resulting concentration of iron (as Fe^3+^) in the iron-rich KOH electrolyte was 5 ppm. KOH electrolyte solutions with intermediate iron contents (*e.g.* 100 ppb) were prepared through dilution from the 18000 ppm Fe^3+^ stock solution. The diluted Fe(NO_3_)_3_ solution was then immediately used in the same way as described for the iron-rich KOH electrolyte. It is important to note that the diluted aqueous (*e.g.* 36 ppm Fe) solutions of Fe(NO_3_)_3_ are not stable and cannot be stored, since Fe^3+^ hydrolyses and eventually precipitates as Fe_2_O_3_ after two or three weeks.^32^ On the other hand, no precipitation of iron was observed from concentrated solutions for over six months.

### Electrochemical measurements

All the results reported in this manuscript are corrected for the uncompensated resistance (*R*_u_, typically around 2.0–2.5 Ω), which was measured through potentiostatic electrochemical impedance spectroscopy (EIS) at 1.43 V *vs.* RHE, 10 mV amplitude, 100 kHz–10 kHz, at the beginning of each experiment. A conditioning period of 60 s at 1.43 V *vs.* RHE was run prior to each EIS measurement, to ensure that Ni(iii) was the predominant nickel species at the electrode surface.

Cyclic voltammetry was measured at a 10 mV s^−1^ scan rate, with a minimum of 3 scans. Only the third scan was taken into account.

Chronopotentiometric tests were carried out for a duration of 72 h at a current of 57 mA with a Ni wire electrode having a length of 1.2 cm as the WE (current density of 300 mA cm^−2^); or at a current of 76 mA with a Ni wire electrode having a length of 0.6 cm as the WE (current density of 800 mA cm^−2^). For the tests at a higher current density (800 mA cm^−2^), a shorter Ni wire (0.6 cm instead of 1.2 cm) was used to limit the total current, and avoid issues related to mass transport limitations and electroosmotic drag of water from one side to the other of the membrane.

The pH was checked before and after each chronopotentiometric test with a pH-meter. No significant difference was observed between the initial and final pH values (*ca.* 13.8–13.9).

The electrochemically active surface area (ECSA) was estimated by following a reported method.^33^ The region between 0.81 and 1.01 V *vs.* RHE was scanned forward and backward with linear sweep voltammetry (LSV) at different scan rates (25 mV s^−1^, 50 mV s^−1^, 100 mV s^−1^, 200 mV s^−1^ and 400 mV s^−1^). At the end of each scan, the potential was maintained for 5 s at the final value (1.01 V *vs.* RHE for the forward scans and 0.81 V *vs.* RHE for the backward scans). The values of the measured current (*I*_m_) at 0.91 V *vs.* RHE in the forward and backward LSV scans were plotted in two graphs, reporting *I*_m_*vs.* scan rate (see Fig. S22†). The average of the modulus of the slopes, obtained from the linear fit of the two plots, gives the double layer capacitance of the sample (*C*_dl_). The *C*_dl_ value was then divided by the specific capacitance^34^ for nickel in 1 M NaOH (*C*_s_ = 25 μF cm^−2^) to obtain the electrochemically active surface area (ECSA = *C*_dl_/*C*_s_).

The reproducibility of our tests was carefully investigated by performing each electrochemical experiment at least in duplicate. A high degree of reproducibility of our results was demonstrated (for example, see Fig. S2† for three chronopotentiometric tests with the regeneration approach in the commercial 1.0 M KOH electrolyte). In the experiments in which the electrolyte was first purified from iron and then defined aliquots of iron were intentionally added, adopting the well-defined procedure described above for the preparation of the electrolyte proved essential to achieve the desired high reproducibility of the results.

### ToF-SIMS analysis

All the ToF-SIMS measurements were conducted with a ToF·SIMS^5^ (IONTOF GmbH, Münster, Germany) time-of-flight secondary ion mass spectrometer. The instrument is equipped with a Cs^+^ gun as the sputtering source and a liquid metal ion gun (Bi) as the analytical source, both mounted at 45° with respect to the sample surface. The time-of-flight mass analyser is perpendicular to the sample surface (Fig. 4a). Before the 2D image acquisitions, the sample surfaces were cleaned with an Ar_5000_^+^ gas cluster ion beam at 10 keV (ion fluence = 2 × 10^13^ ions per cm^2^) in order to remove possible organic contaminations. 2-D images were recorded by using a pulsed beam of Bi_3_^++^ (energy: 60 keV). An AC current of 0.001 pA was used with the analytical burst mode (1 pulse selected with the sine blanker). A raster of 512 × 512 pixels over an area of 150 μm × 150 μm was selected. The cycle time was 100 μs. Lateral resolution of 0.3 μm and mass resolution *m*/Δ*m* > 2800 at 58 *m*/*z* (corresponding to ^58^Ni^+^) were maintained for the data acquisition. Surface measurements were carried out with a ‘non-interlaced’ mode (analysis: 1.64 s; sputtering: 1.64 s; pause: 2.7 s). The Cs^+^ ion source was operated at 1 keV with a DC current of 70 nA. For depth profiling, the focussed Cs^+^-beam of primary ions was rastered on a square of 128 × 128 data points covering an area of 450 μm x 450 μm. A pulsed beam of 30 keV Bi^+^ ions (AC current of 1.4 pA) was employed to provide mass spectra from a square of 128 × 128 data points covering an area of 150 μm × 150 μm in the centre of the sputter crater. The mass resolution *m*/Δ*m* was >6000 at 58 *m*/*z* (corresponding to ^58^Ni^+^). The cycle time was 100 μs. The depth profiling distance was calibrated by sputtering a mirror-like polished 1.6 mm thick nickel plate (99.0% nickel, Goodfellow) for a certain amount of time and then measuring the crater with a stylus profilometer (DektakXT, Bruker).

Each of the surface measurement data reported in Fig. 4c and 5a is the average of the results of the analysis of four different spots, each with an area of 150 μm × 150 μm, randomly chosen on the wires. The error bars are the standard deviation of the four results.

For the depth profile analysis, selected samples were analysed by measuring the depth profile for two different, randomly chosen spots. In such cases, we reported the average of the two depth profile plots, which was calculated using the OriginLab software. Since the data sets are characterised by slightly different sampling rates, calculating the average required introducing a tolerance value on the *x*-axis. In each case, the minimum possible tolerance was used (0.1 for “fully active” in Fig. 4d, 0.001 for “before deactivation” in Fig. 4d, 0.6 for “without regeneration” in Fig. 5b and 0.2 for “before regeneration” in Fig. 5b). It is worth noting that while the depth profile of the two different spots showed some deviation from each other, in all cases the conclusions drawn in the discussion would be the same if only one of the two data sets was used.

### XPS analysis

All the samples for XPS analysis were dipped in MilliQ water for a few seconds (<10 s) at the end of the experiments in order to remove excess KOH. This step was kept short to prevent loss of iron from the surface. A fresh Ni wire, pretreated with 15 min sonication in ethanol followed by 10 min sonication in MilliQ water, was used as a reference. The XPS analysis was performed using a Surface Science Instruments SSX-100 ESCA instrument with a monochromatic Al Kα X-ray source (*hν* = 1486.6 eV). The pressure in the measurement chamber was maintained below 8 × 10^−9^ mbar during data acquisition. The electron takeoff angle with respect to the surface normal was 37°. The diameter of the analysed area was 1000 μm; the energy resolution was 1.26 eV (or 1.67 eV for a broad survey scan). XPS spectra were analysed using the least-squares curve fitting program CasaXPS, developed by Casa Software Ltd, and included a Shirley baseline subtraction and a peak deconvolution using a linear combination of Gaussian and Lorentzian functions, taking into account the experimental resolution. The spectra were fitted with a minimum number of peaks consistent with the structure of the surface, a Shirley background and a GL(30) peak shape. Binding energies of isolated peaks have an uncertainty of ±0.3 eV. Binding energies were referenced to the C1s photoemission peak originating from adventitious carbon (C–C/C=C) at a binding energy of 285.3 eV. All the measurements are available in Fig. S3–S8.†

### ICP-OES analysis

Approximately 340 mg of pristine nickel wire (99.995% Ni, 0.5 mm diameter, Puratronic, AlfaAesar) were immersed in 7 ml of 65% nitric acid aqueous solution. After stirring for 2 weeks at room temperature, digestion was completed. Double distilled water was added to the solutions until reaching a final volume of 20 mL. These solutions were prepared in duplicate. The ICP-OES measurements were performed using an Optima 70 000 utilising a calibration mixture 11 355 from Sigma Aldrich in the range of 1.5 to 10 ppm. The concentrations of iron and cobalt (the latter being an impurity in the Ni wire) were calculated by means of the calibration curves. The average and standard deviations were calculated from the duplicated measurements. The results obtained with a wavelength of 228.6 nm for cobalt and 238.2 nm for iron were used for this purpose and are reported in Table S1.†

### SEM images

Imaging of the samples was performed using an FEI Nova NanoSEM 650 at 30 kV. A custom-made sample holder was used, consisting of an electrically conductive clamp soldered on a copper wire. The part with the copper wire was placed in the stage of the SEM. The part with the conductive clamp was used to hold the Ni wire electrodes to be analysed.

## Conflicts of interest

There are no conflicts to declare.

## Supplementary Material

GC-027-D5GC00114E-s001

## Data Availability

Data for this article are available either in the ESI† or, in the case of the raw data, at DataverseNL with the link https://doi.org/10.34894/XW7RK6.
