# Supplementary material for: Regeneration of iron species for high and stable activity of nickel electrodes in the oxygen evolution reaction
Source: Green Chem. 2025 Apr 11;27(28):8505–16. doi: 10.1039/d5gc00114e (PMC12053971; doi:10.1039/d5gc00114e)
Supplement: GC-027-D5GC00114E-s001 [file GC-027-D5GC00114E-s001.pdf]

Supplementary Information (SI) for Green Chemistry.
This journal is © The Royal Society of Chemistry 2025

Supplementary Information

Regeneration of iron species for high and stable activity of nickel electrodes in the oxygen evolution reaction

Stefano Poli[a], Claude Poleunis[b], Matteo Miola[a], Dominic Gerlach[c], Petra Rudolf[c], Arnaud Delcorte[b], Hans Lammers[d], Matheus T. de Groot[e,f], Dulce M. Morales[a], Paolo P. Pescarmona[a,*]

[a]*Chemical Engineering group, Engineering and Technology institute Groningen (ENTEG), University of Groningen, The Netherlands*

[b]*Institute of Condensed Matter and Nanosciences - Bio & Soft Matter, Surface Characterisation, Université Catholique de Louvain, Louvain-la-Neuve, Belgium*

[c]*Zernike Institute for Advanced Materials, University of Groningen, The Netherlands*

[d]*Nobian, Van Asch van Wijckstraat 53, Amersfoort, The Netherlands*

[e]*HyCC B.V., Van Asch van Wijckstraat 53, Amersfoort, The Netherlands*

[f]*Department of Chemical Engineering and Chemistry, Sustainable Process Engineering Group, Eindhoven University of Technology, the Netherlands*

corresponding author: p.p.pescarmona@rug.nl

Supplementary Note 1 The power saved using our regeneration strategy during an experiment in commercial KOH with and without regeneration steps (from Fig. 1) was estimated by first assessing the power consumption to reach a total current of 0.057 A (electrode geometric surface area 0.19 cm^2, thus 300 mA cm^{-2}).

$$P = V_{measured} \cdot 0.057\ A$$

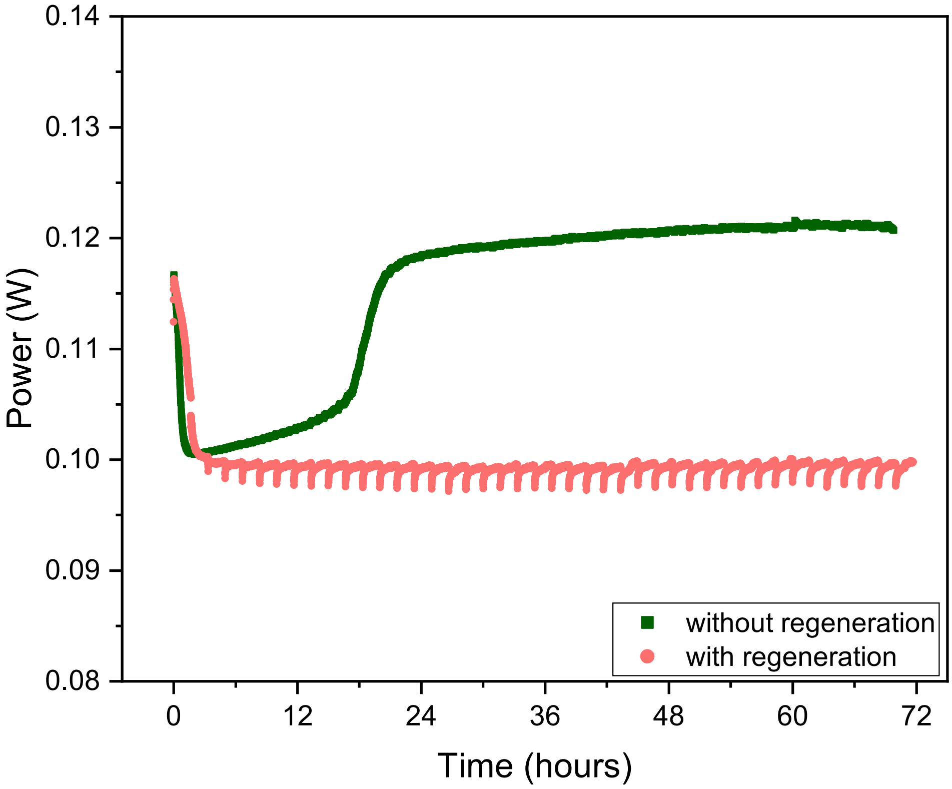

The electrical energy utilised in each experiment is obtained by integrating the area under the curves (by integration of the plots in Origin).

$$E_{without\ regeneration} = 8.05\ Wh$$

$$E_{with\ regeneration} = 6.96\ Wh$$

$$E_{saved}(\%) = \frac{E_{without\ regeneration} - E_{with\ regeneration}}{E_{without\ regeneration}} \cdot 100 = \frac{8.05 - 6.96}{8.05} \cdot 100 = 13.5\ \%$$

The resulting value was rounded up to 14% in the main text and it is relative to the full 72 h of experiment, including the activation and deactivation period of the sample without regeneration.

An estimation of the long-term energy saving can be done by excluding the first 24 h of the experiments in the comparison, since real-life electrolysers are meant to operate for months, thus making the energy saved during activation and deactivation period of the sample without regeneration irrelevant.

The electrical energy saved in the range from 24 to 72 h of the experiments amounts to:

$$E_{without\ regeneration} = 5.65\ Wh$$

$$E_{with\ regeneration} = 4.63\ Wh$$

$$E_{saved}(\%) = \frac{E_{without\ regeneration} - E_{with\ regeneration}}{E_{without\ regeneration}} \cdot 100 = \frac{5.65 - 4.63}{5.65} \cdot 100 = 18.0\ \%$$

The resulting value of 18% was reported in the main text.

Supplementary Note 2 The experiments were carried out with a 1 M KOH Hg/HgO reference electrode with potential of 0.098 V vs SHE.

The values of potential (*V*) in all the plots have been converted to RHE with the following formula:

$$V_{vs\ RHE} = V_{measured} + 0.098\ V + 0.059\ V{\cdot}pH$$, where pH = 13.8

Supplementary Note 3 The depth-profiling analysis by SIMS-TOF was calibrated by sputtering a mirror-like nickel foil (99.0% Nickel Foil 1.6mm, Goodfellow) for a certain amount of time and then measuring the depth of the crater with a profilometer. This operation is crucial in order to have an accurate depth-profile information, since the number of ions extracted from the samples is strongly related to the type of material. For this purpose, we used a nickel foil as this is the closest approximation to our nickel wires, which mainly consist of nickel. The of SIMS-TOF output is reported in "fluence" (ions cm^{-2}), which is the amount of ions extracted from unit surface area.

The milling of the foil resulted in $1.3{\cdot}10^{18}$ ions cm^{-2}, which corresponds to a crater with a 800 nm depth according to the profilometer.

Therefore, we can estimate the *milling rate* as:

$$\frac{800\ nm}{1.3 \cdot 10^{18}\ ions\ cm^{-2}} = 6.15{\cdot}10^{-16}\frac{nm\ cm^2}{ions}$$

We can use the milling rate to convert the fluence information from our samples into depth in nm:

$$Depth\ (nm) = fluence\left[\frac{ions}{cm^2}\right] \cdot milling\ rate\left[\frac{nm\ cm^2}{ions}\right]$$

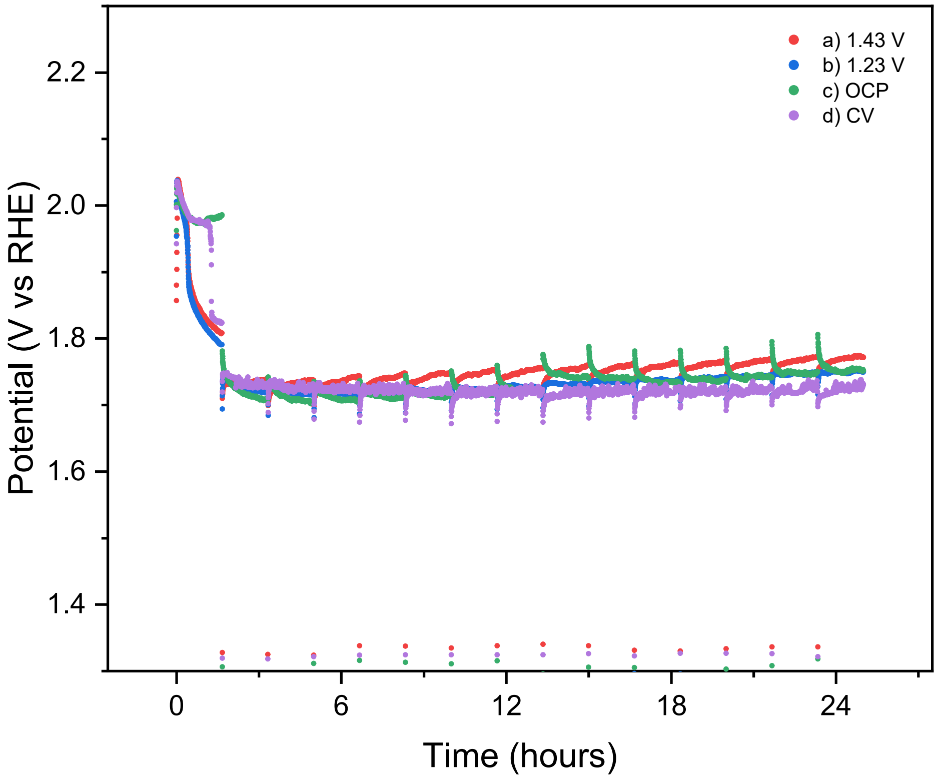

Fig. S1 Preliminary investigation of the regeneration at different conditions, in unpurified 1.0 M commercial KOH (Supelco, declared iron content < 0.0005 %) at 30 °C, at 300 mA cm^{-2}. The potential was held for 13 minutes at (a) 1.43 V vs RHE (after Ni(II)/Ni(III) redox peak), (b) 1.23 V vs RHE (before Ni(II)/Ni(III) redox peak), and (c) at the Open circuit potential (OCP). (d) Cyclic voltammetry at each regeneration step between 1.23 V and 1.43 V, 40 cycles, 20 mV s^{-1} scan rate (total duration of the step ca. 13 minutes). All the curves are iR compensated.

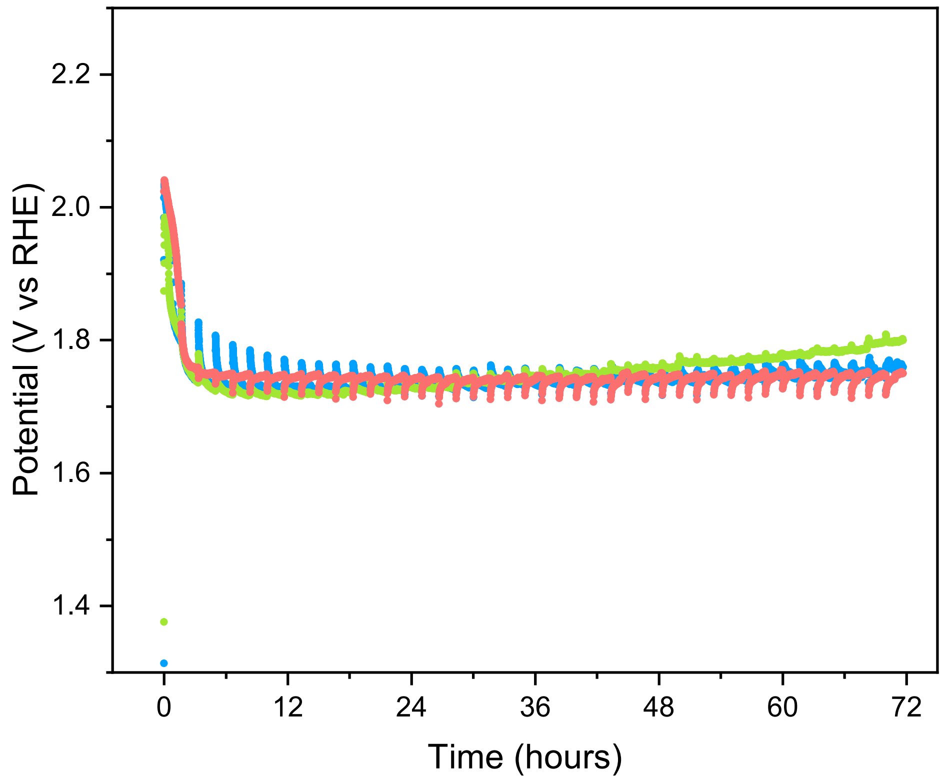

Fig. S2 Reproducibility of the chronopotentiometric tests of three different experiments at 300 mA cm^{-2} with regeneration steps (1.47 V for 100 s every 100 min of electrolysis) in 1.0 M commercial KOH (Supelco, declared iron content < 0.0005 %) at 30 °C. All the curves are iR compensated.

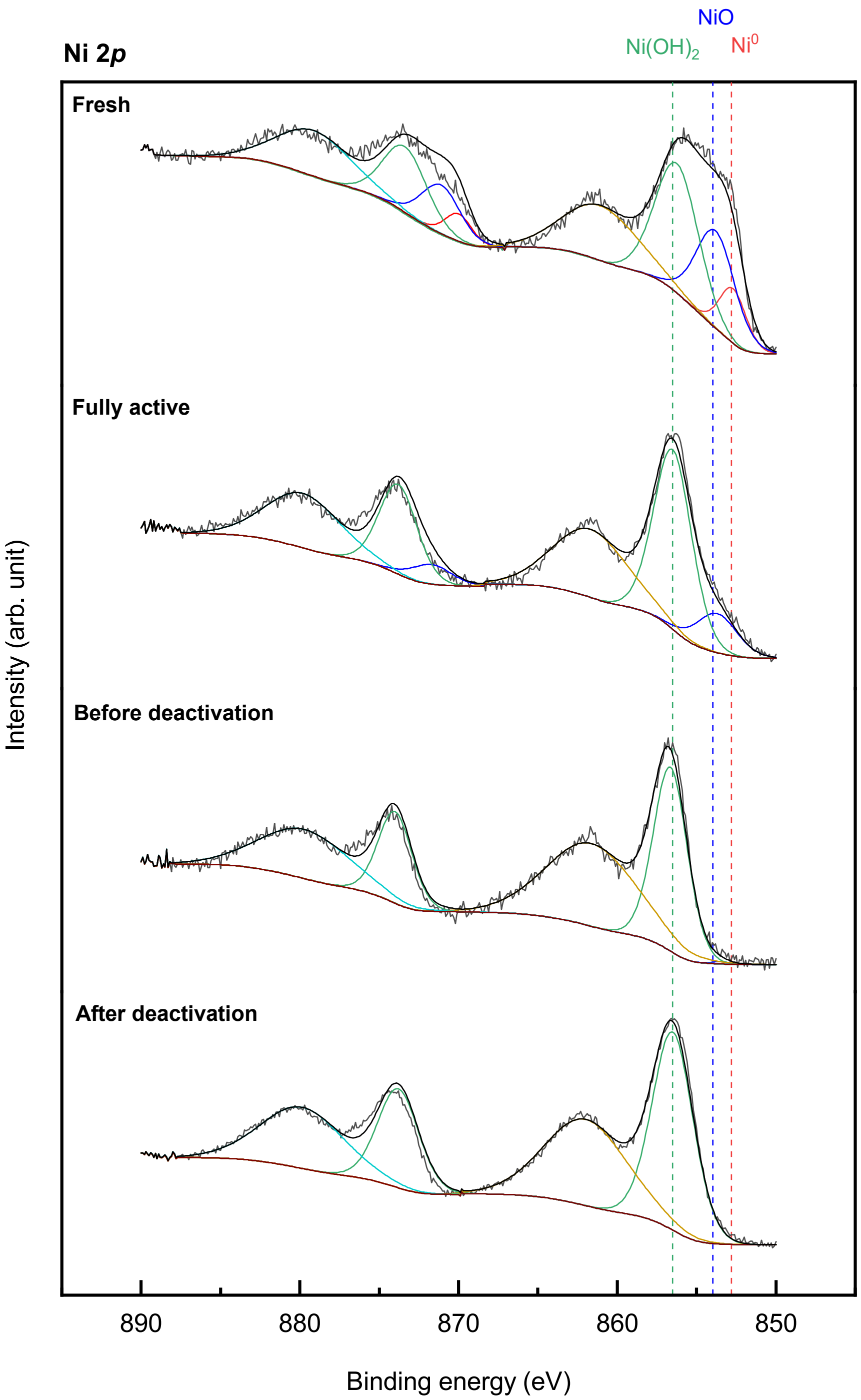

Fig. S3 XPS spectra of the Ni 2*p* core level region of the wires: fresh, fully active, just before deactivation and after deactivation. These data show that Ni^0 and NiO are progressively converted into $Ni(OH)_2$ and/or NiOOH during electrolysis.

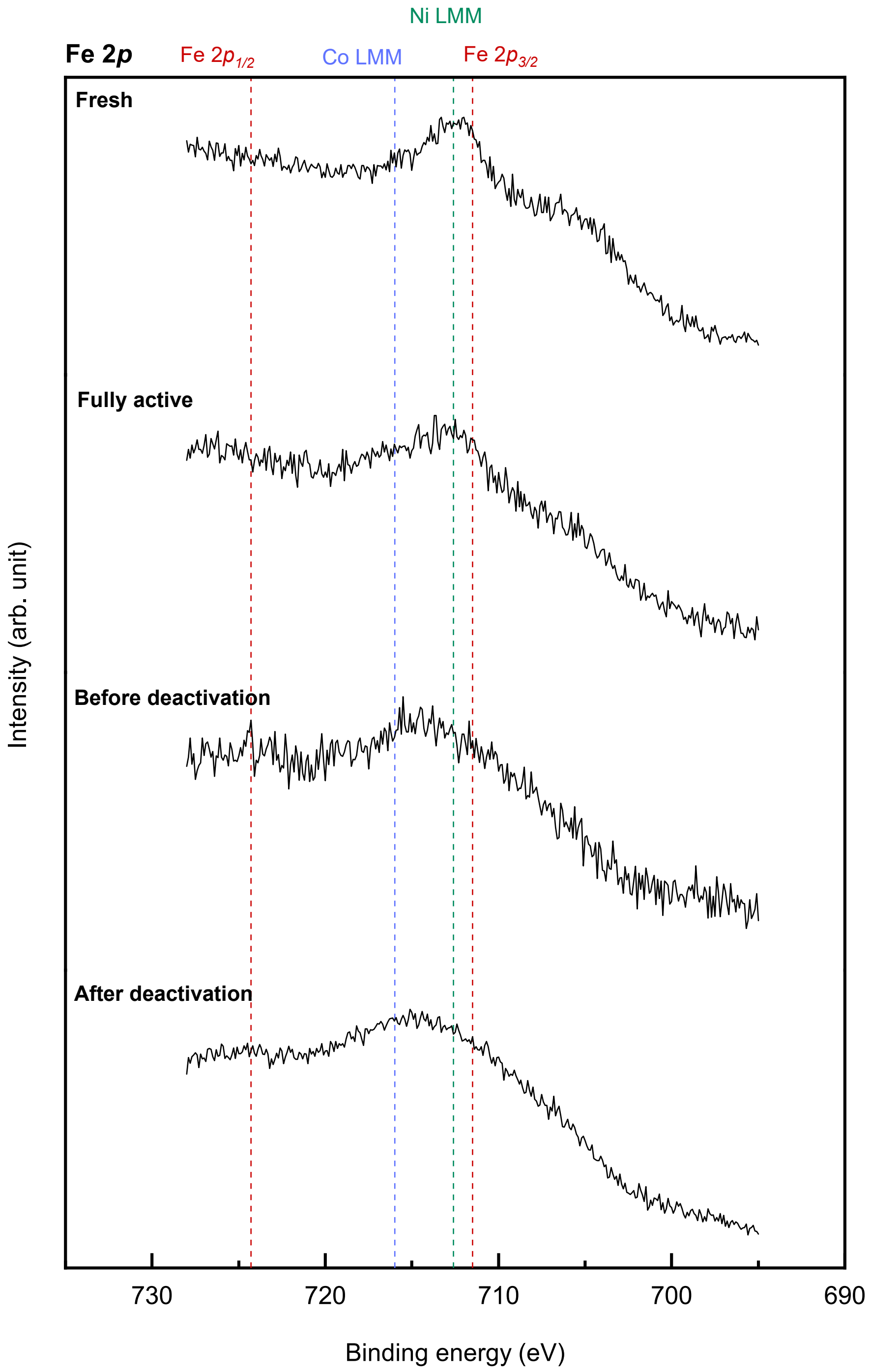

Fig. S4 XPS spectra of the Fe 2*p* core level region of the wires: fresh, fully active, just before deactivation and after deactivation. The Fe 2*p* signals are not visible due to the very low iron content and the presence of overlapping Ni LMM and Co LMM signals (Al Kα X-ray source was used).

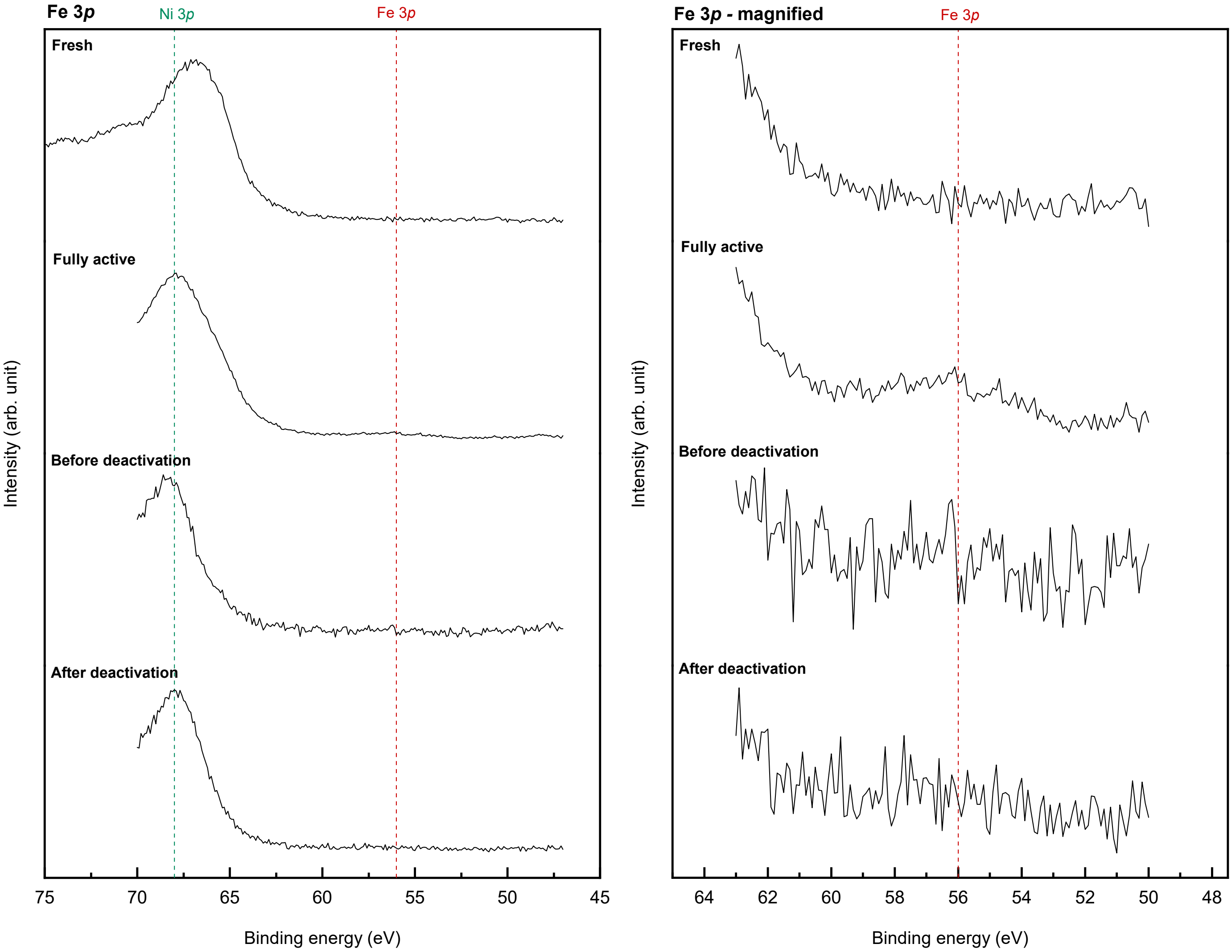

Fig. S5 XPS spectra of the Fe 3*p* core level region of the wires: fresh (160 scans), fully active (160 scans), just before deactivation (80 scans) and after deactivation (80 scans). The Fe 3*p* expected to be peaked at 56 eV is barely visible due to the very low iron content of the samples.

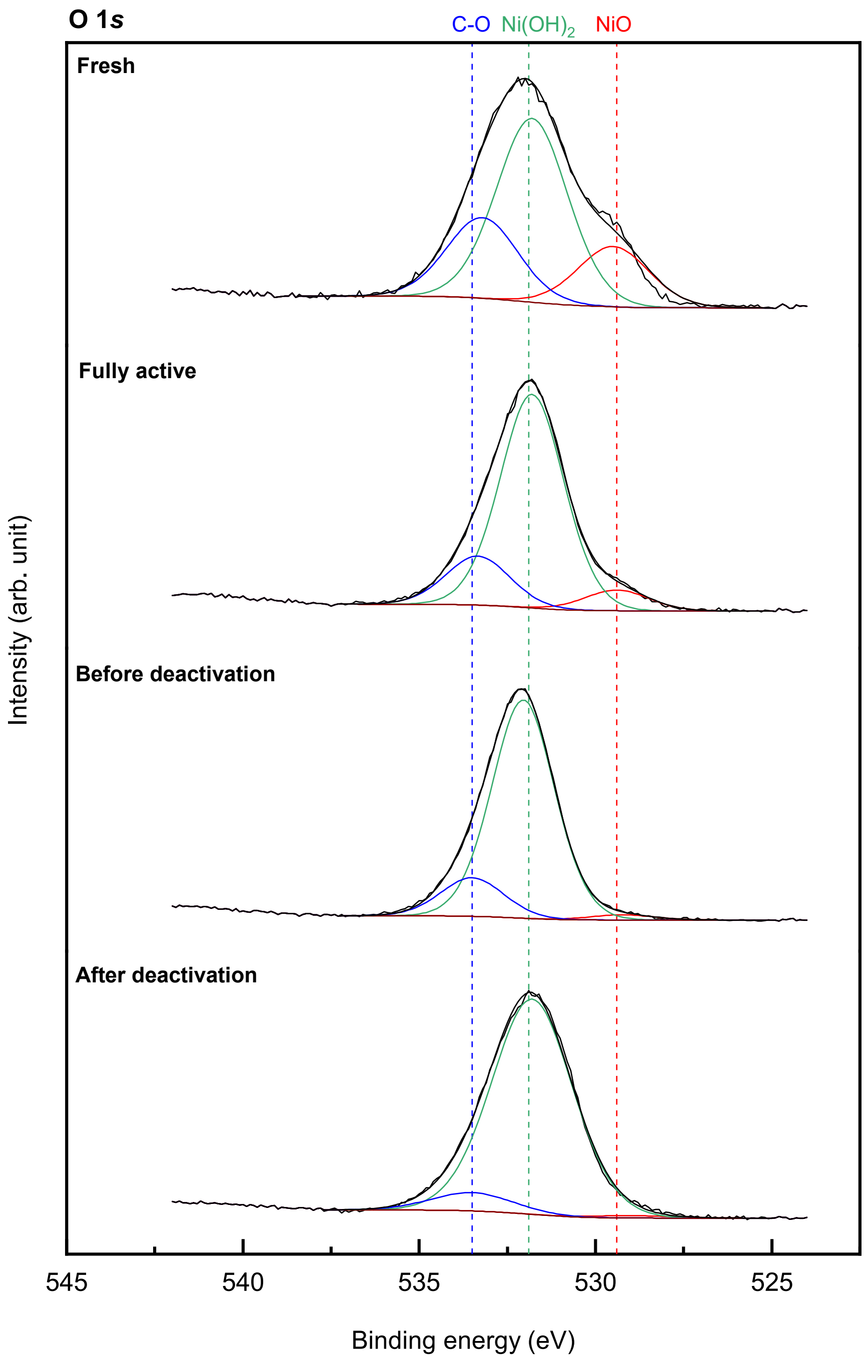

Fig. S6 XPS spectra of the O 1*s* core level region of the wires: fresh, fully active, just before deactivation and after deactivation, and corresponding fits. These data are in agreement with the gradual conversion of NiO into $Ni(OH)_2$ and/or NiOOH during electrolysis (see Fig. S3).

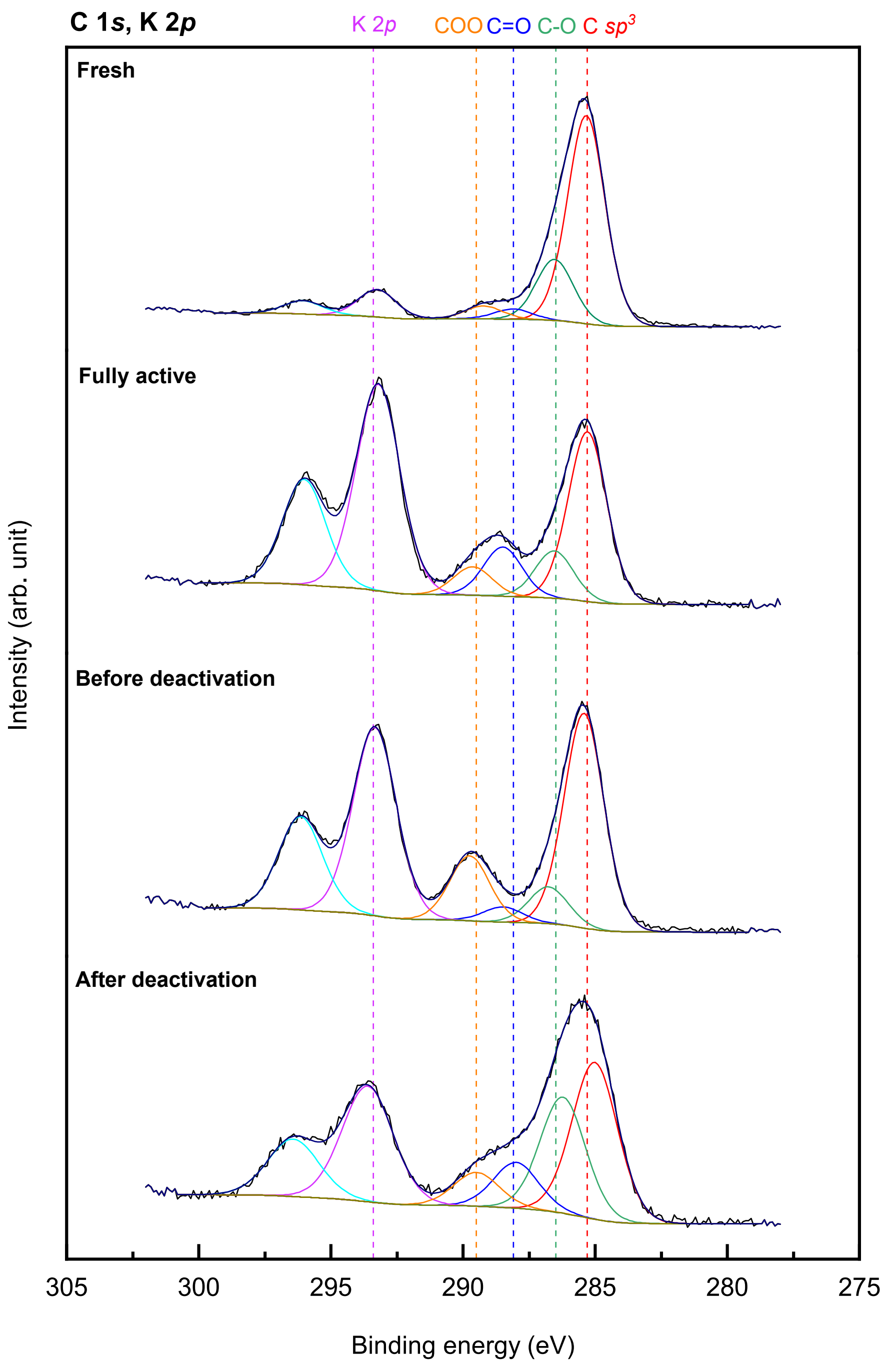

Fig. S7 XPS spectra of the C 1*s* and K 2*p* core level region of the wires: fresh, fully active, just before deactivation and after deactivation. The carbon content is probably due to contamination with air. Carbonates may be stem from the dissolution of CO_2 present in air into the KOH solution during electrolysis. The wires were moderately rinsed with MilliQ water after electrolysis; however, they still show the presence of potassium from the aqueous KOH electrolyte. It is possible that part of K^+ and CO_3^{2-} ions are intercalated in the layered structure o $Ni(OH)_2$ and/or NiOOH[1].

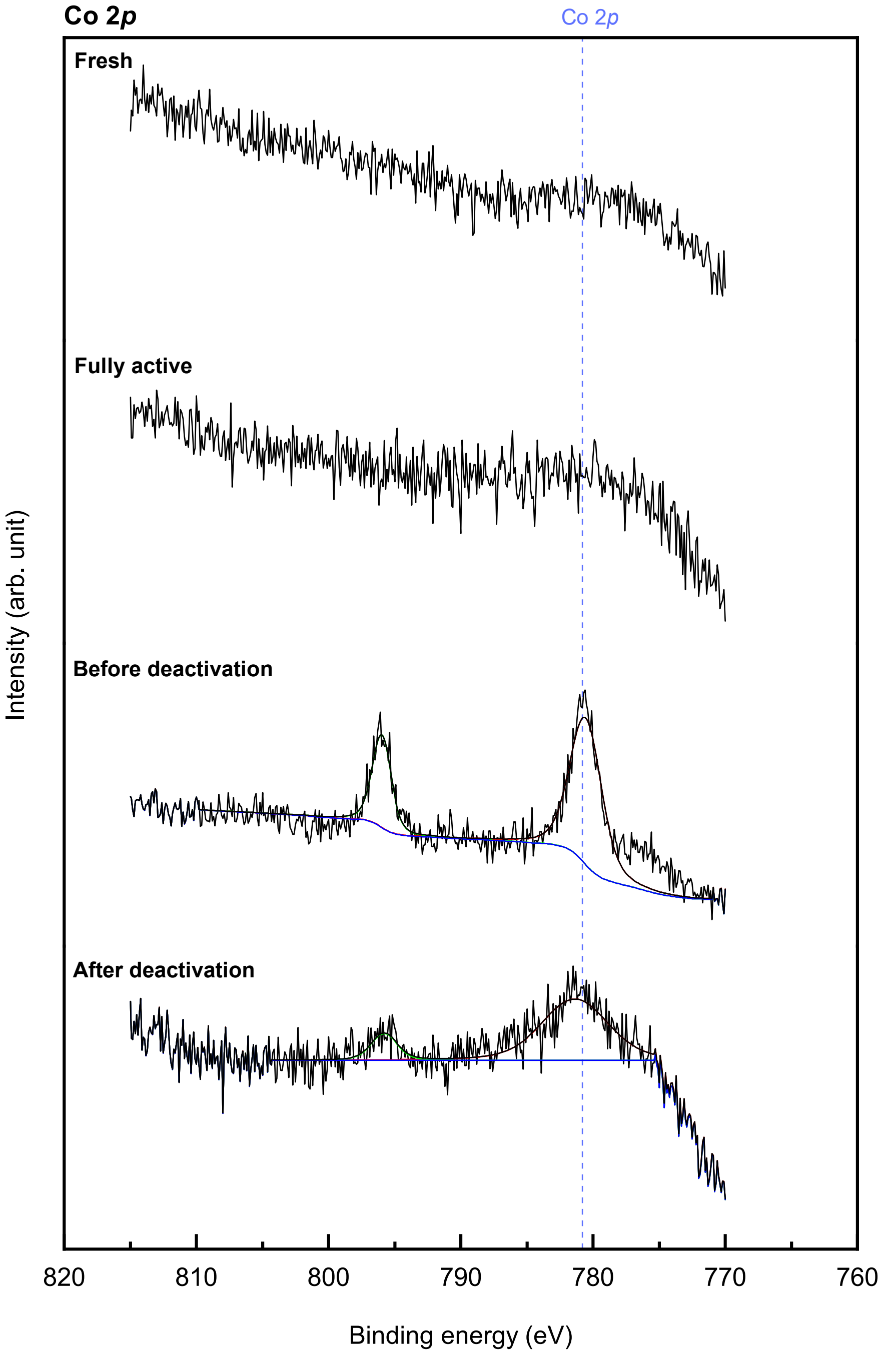

Fig. S8 XPS spectra of the Co 2*p* core level region of the wires: fresh, fully active, just before deactivation and after deactivation. The data show the presence of cobalt in the electrodes. ICP-OES analysis (Supplementary Table 1) of the fresh Ni wires proves that the observed Co is an impurity of the wire and is not introduced from the electrolyte during the experiments.

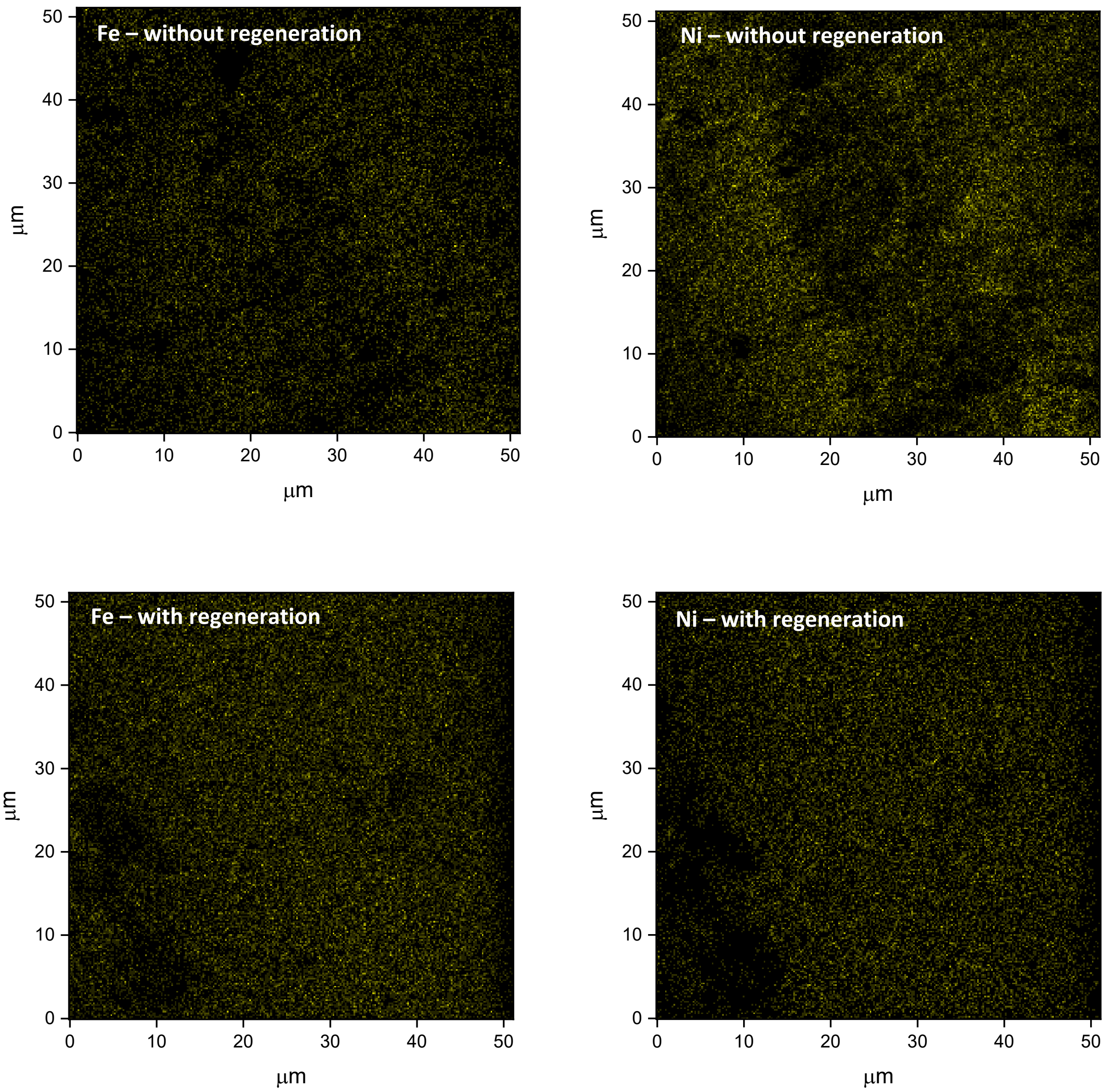

Fig. S9 ToF-SIMS mapping of the surface of the same two electrodes after the chronopotentiometric test with the iron-rich electrolyte, with and without regeneration. Yellow is the total sum of positively charged iron-containing fragments of iron ($^{54}Fe^+$, Fe^+, FeH^+, $FeOH^+$) and nickel (Ni^+, NiH^+, $^{60}Ni^+$, $^{60}NiH^+$, $^{60}NiH_2^+$).

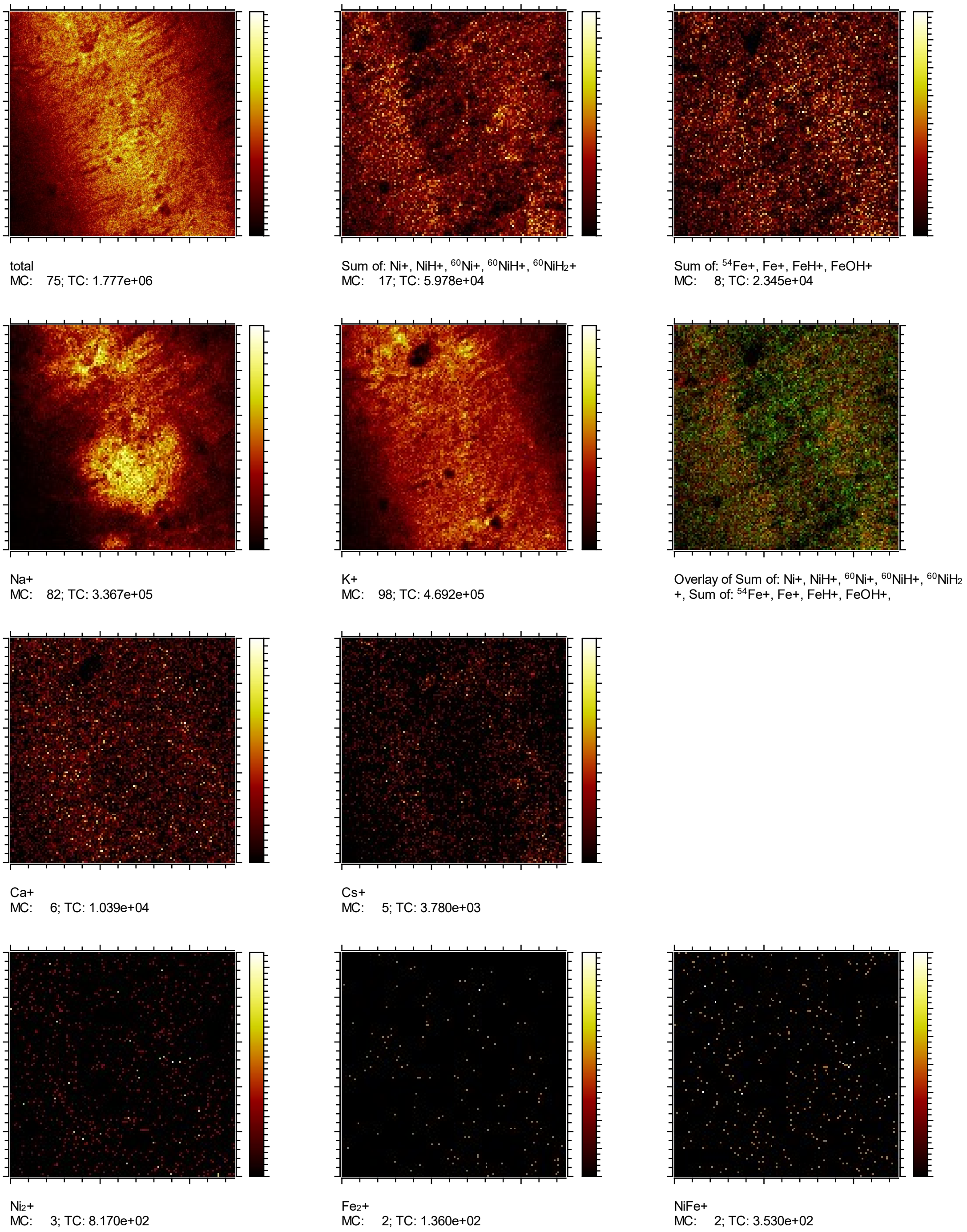

Fig. S10 ToF-SIMS mapping of several elements of the surface of the electrode tested in iron-rich electrolyte for 72 h without regeneration. Cs^{+} is an impurity introduced during the previous ToF-SIMS measurement (depth-profiling).

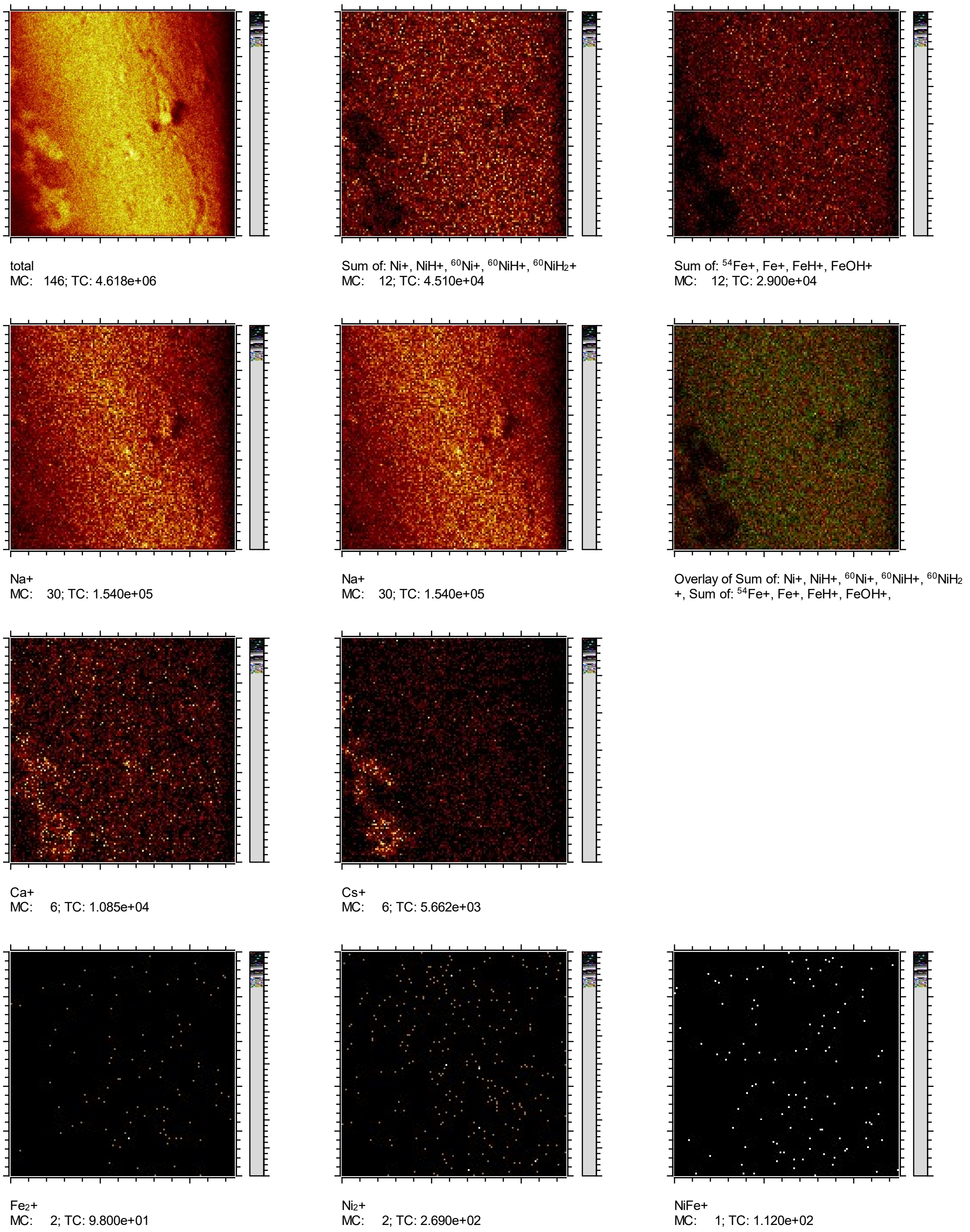

Fig. S11 ToF-SIMS mapping of several elements of the surface of the electrode tested in iron-rich electrolyte for 72 h with regeneration. Cs^+ is an impurity introduced during the previous ToF-SIMS measurement (depth-profiling).

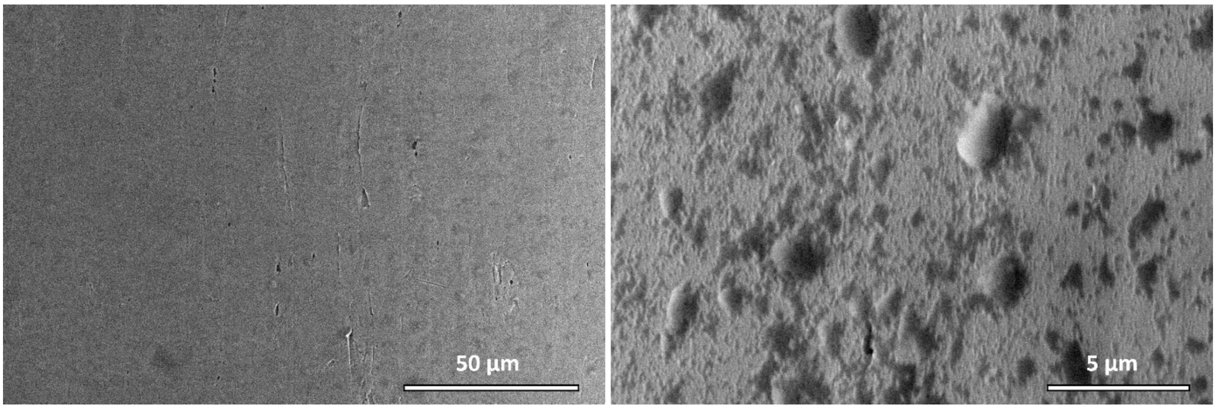

Fig. S12 SEM image collected at 18 kV acceleration voltage. Sample “fully active”, tested at 100 ppb Fe in 1.0 M KOH at 300 mA cm^{-2}.

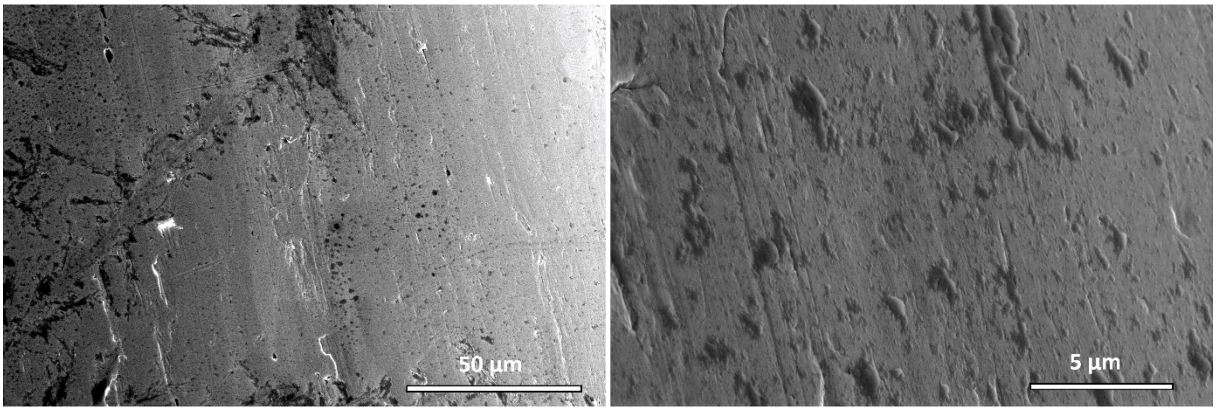

Fig. S13 SEM image collected at 18 kV acceleration voltage. Sample “before deactivation”, tested at 100 ppb Fe in 1.0 M KOH at 300 mA cm^{-2}.

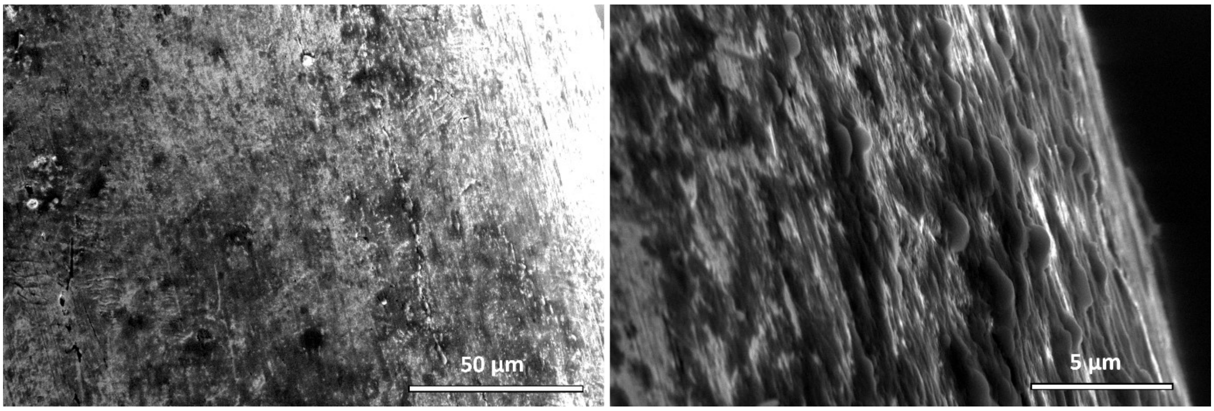

Fig. S14 SEM image collected at 18 kV acceleration voltage. Sample “after deactivation”, tested at 100 ppb Fe in 1.0 M KOH at 300 mA cm^{-2}.

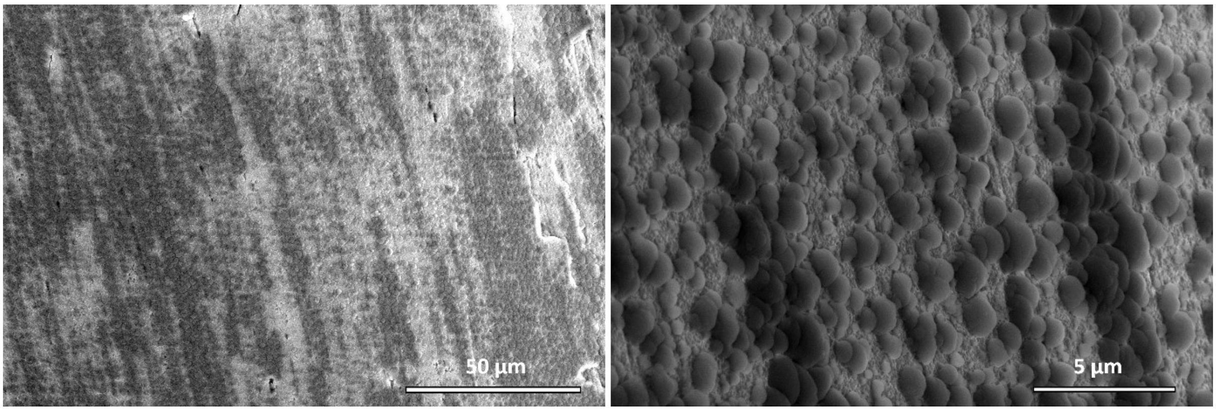

Fig. S15 SEM image collected at 18 kV acceleration voltage. Sample tested in iron-lean 1.0 KOH at 300 mA cm^{-2} for 72 h, without regeneration.

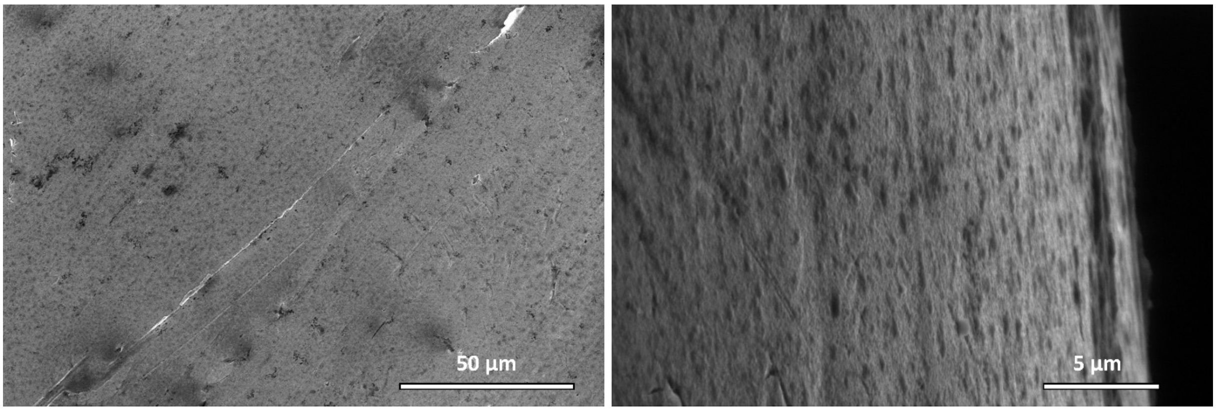

Fig. S16 SEM image collected at 18 kV acceleration voltage. Sample tested at 5 ppm Fe in 1.0 KOH at 300 mA cm^{-2} for 72 h, without regeneration.

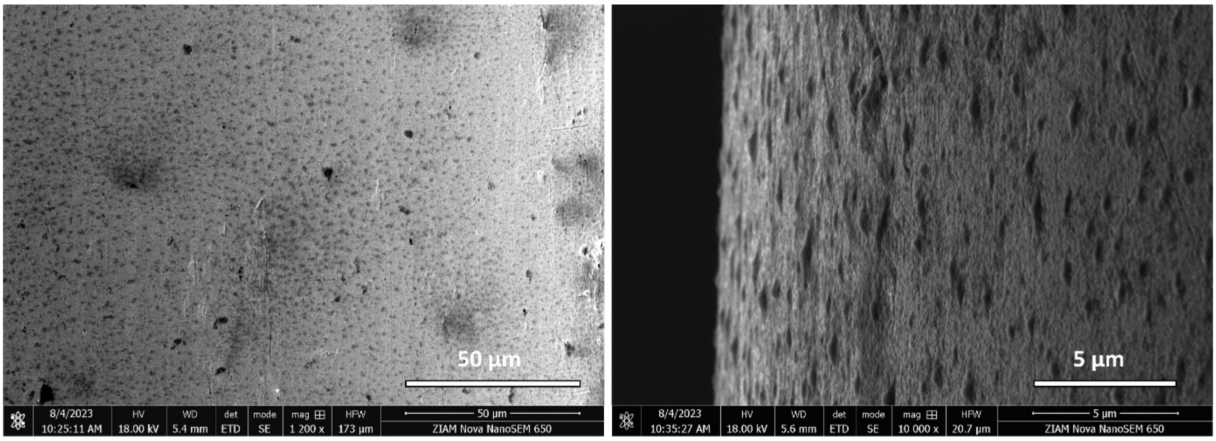

Fig. S17 SEM image collected at 18 kV acceleration voltage. Sample tested at 5 ppm Fe in 1.0 KOH at 300 mA cm^{-2} for 72 h, with regeneration.

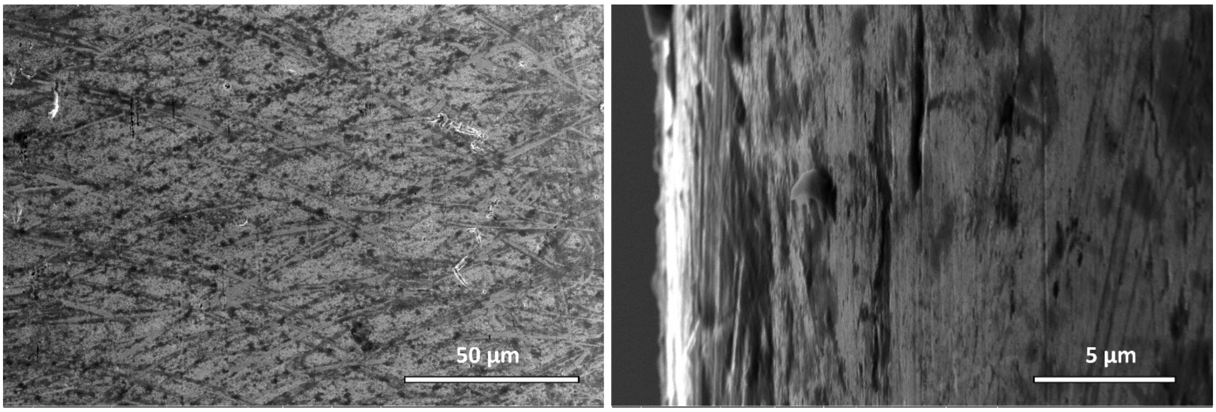

Fig. S18 SEM image collected at 18 kV acceleration voltage. Nickel wire tested in 100 ppb Fe with regeneration steps for 72 h at 300 mA cm^{-2}. The scarring on the surface is attributed to the handling when inserted and removed from the ToF-SIMS equipment (the SEM images in Fig. S12-17 were measured before the samples were analyzed with ToF-SIMS).

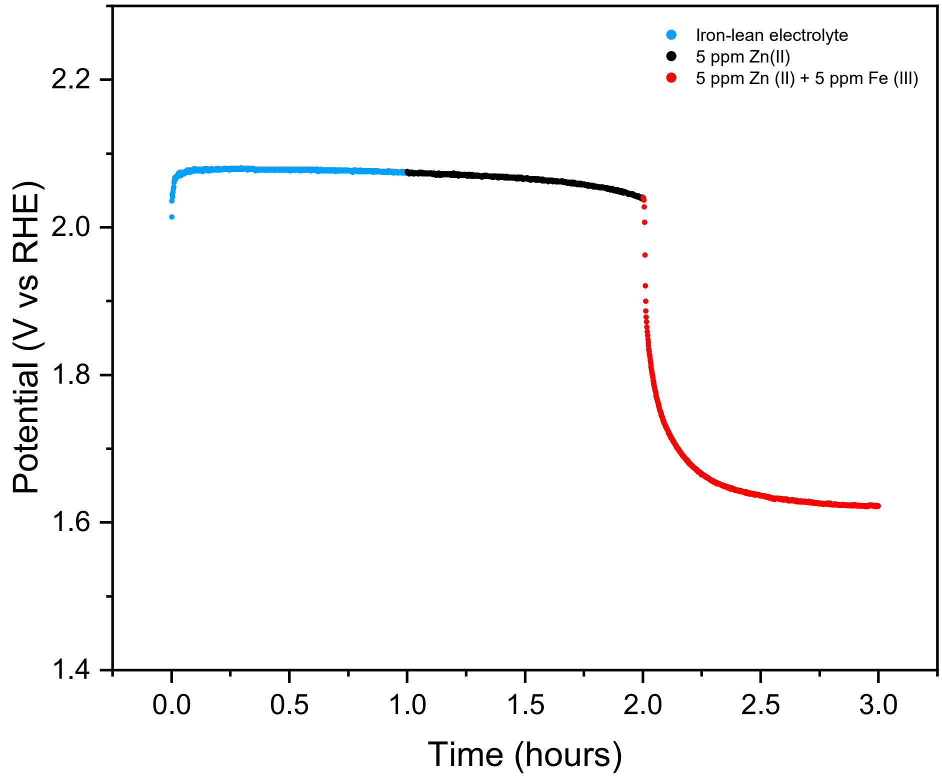

Fig. S19 Effect of the addition of 5 ppm of Zn(II) and of 5 ppm of Fe(III) to purified 1.0 M commercial KOH (Supelco, declared iron content < 0.0005 %) at 30 °C, at 300 mA cm^{-2}. The metals were added in sequential order: ca. 5 ppm of Zn (as $ZnSO_4$, for analysis, EMSURE) was added after 1 h of electrolysis in iron-lean electrolyte. Then, after another 1 h of electrolysis, ca. 5 ppm of Fe (III) were also added.

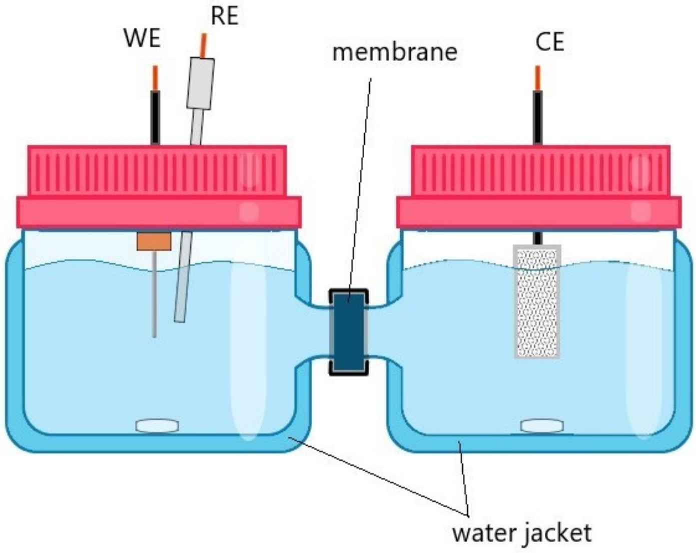

Fig. S20 Schematic representation of the electrochemical cell used in this work. The half-cell containers are made of glass. Each half-cell is place over a magnetic stirrer. Teflon-coated magnetic stirrers were used during the experiments. The working electrode (nickel wire) was held by a copper clamp that was standing positioned the cell but outside the electrolyte solution. The total length of the nickel wire was ca. 2 cm, of which 1.2 cm were immersed in the electrolyte for the electrochemical tests. The copper clamp was accurately wrapped with several layers of parafilm prior each experiment to prevent the humidity to directly condense on it during the experiment.

To clean the electrochemical cell, the setup was disassembled and each component (including the magnetic stirrers) was rinsed with high purity aqueous HCl (Sigma-Aldrich, 37 wt. % in H_2O, 99.999% trace metals basis) and subsequently by abundant MilliQ water (16.2 MΩ). This procedure was followed every time we were moving from an iron-containing experiment to one with smaller concentration (e.g. 5 ppm Fe $\rightarrow$ 100 ppb Fe or 100 ppb Fe $\rightarrow$ iron-lean). During this procedure, the membrane (Selemion AHO) was replaced with a new one (the new membranes were activated for 1 h in commercial 1M KOH before use). In any other situation, the cell was simply rinsed with copious amount of MilliQ water between the experiments.

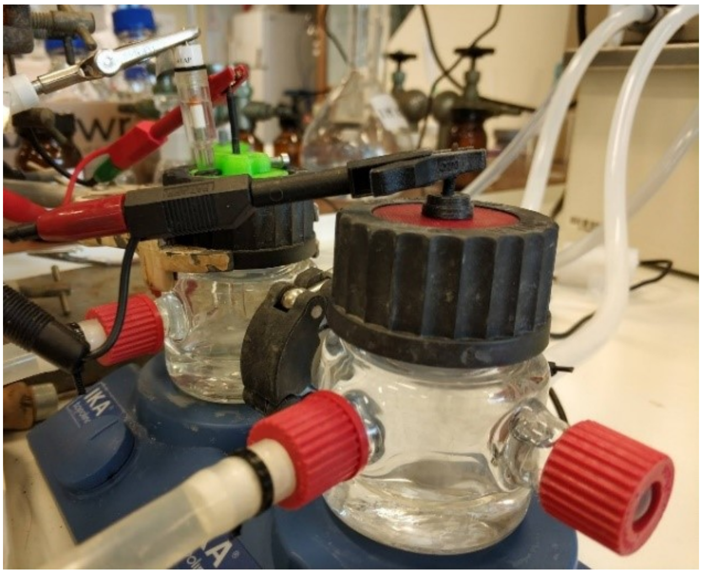

Fig. S21 Picture of the electrochemical set-up used in this work.

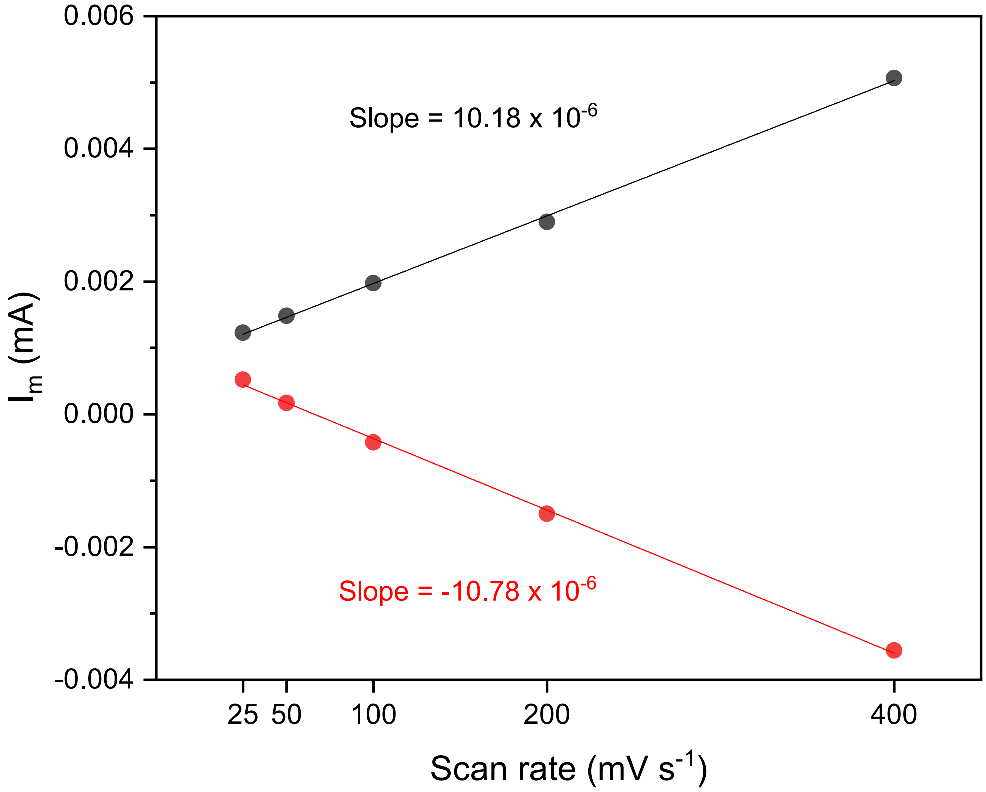

Fig. S22 The measured current I_m is plotted versus the scan rate for the LSV from 0.81 to 1.01 V vs RHE (in black) and for the LSV from 1.01 to 0.81 V vs RHE (in red). The values were collected at the potential 0.91 V vs RHE. The average of the modulus of the slopes corresponds to the value of the double layer capacitance (C_{dl}) of a fresh Ni wire (A_{geo} = 0.19 cm^2) immersed into 1 M KOH electrolyte. C_{dl} = 10.48 µF.

Table S1 ICP-OES measurements as of fresh Ni wire (in duplicate, ca. 340 mg per sample).

Element	Amount (ppm)	σ (standard deviation)
**Co**	151	6
**Fe**	5	1

Prior to the analysis, the fresh Ni wires were cut into small pieces with stainless steel pliers in order to facilitate the digestion. This might have introduced Fe impurities, implying that the iron content reported here is likely to be an overestimate.

References

1. Hall, D. S., Lockwood, D. J., Bock, C. & MacDougall, B. R. Nickel hydroxides and related materials: A review of their structures, synthesis and properties. *Proceedings of the Royal Society A: Mathematical, Physical and Engineering Sciences* **471**, (2015).
